# Cytotoxic Secondary Metabolites Isolated from the Marine Alga-Associated Fungus *Penicillium chrysogenum* LD-201810

**DOI:** 10.3390/md18050276

**Published:** 2020-05-22

**Authors:** Lin-Lin Jiang, Jin-Xiu Tang, Yong-Heng Bo, You-Zhi Li, Tao Feng, Hong-Wei Zhu, Xin Yu, Xing-Xiao Zhang, Jian-Long Zhang, Weiyi Wang

**Affiliations:** 1 School of Life Sciences, Ludong University, Yantai 264025, China; linlinjiang1986@163.com (L.-L.J.); TJX19950209@163.com (J.-X.T.); hngwzhu@outlook.com (H.-W.Z.); yuxinzghn@163.com (X.Y.); 2 Shandong Provincial Key Laboratory of Quality Safty Monitoring and Risk Assessment for Animal Products, Ji’nan 250022, China; yongheng1980@163.com (Y.-H.B.); liyouzhi2009@126.com (Y.-Z.L.); fengtaojn2019@163.com (T.F.); 3 Yantai Key Laboratory of Animal Pathogenetic Microbiology and Immunology, Yantai 264025, China; 4 Key Laboratory of Marine Biogenetic Resources, Third Institute of Oceanography, Ministry of Natural Resources, Xiamen 361005, China

**Keywords:** alga, marine-derived fungus, *Penicillium chrysogenum*, polyketide, hydroxyphenylacetic acid, cytotoxicity

## Abstract

A new pentaketide derivative, penilactonol A (**1**), and two new hydroxyphenylacetic acid derivatives, (2’*R*)-stachyline B (**2**) and (2’*R*)-westerdijkin A (**3**), together with five known metabolites, bisabolane-type sesquiterpenoids **4**–**6** and meroterpenoids **7** and **8**, were isolated from the solid culture of a marine alga-associated fungus *Penicillium chrysogenum* LD-201810. Their structures were elucidated based on extensive spectroscopic analyses, including 1D/2D NMR and high resolution electrospray ionization mass spectra (HRESIMS). The absolute configurations of the stereogenic carbons in **1** were determined by the (Mo_2_(OAc)_4_)-induced circular dichroism (CD) and comparison of the calculated and experimental electronic circular dichroism (ECD) spectra, while the absolute configuration of the stereogenic carbon in **2** was established using single-crystal X-ray diffraction analysis. Compounds **2** and **3** adapt the 2’*R*-configuration as compared to known hydroxyphenylacetic acid-derived and *O*-prenylated natural products. The cytotoxicity of **1**–**8** against human carcinoma cell lines (A549, BT-549, HeLa, HepG2, MCF-7, and THP-1) was evaluated. Compound **3** exhibited cytotoxicity to the HepG2 cell line with an IC_50_ value of 22.0 μM. Furthermore, **5** showed considerable activities against A549 and THP-1 cell lines with IC_50_ values of 21.2 and 18.2 μM, respectively.

## 1. Introduction

Microorganisms belonging to marine ecosystems are diverse both taxonomically and biologically [1,2,3]. These microorganisms developed unique metabolic pathways to overcome the extreme temperature, nutrient scarcity, high salinity, and ultraviolet radiation [4,5]. As one of the most prevalent biocenoses in marine ecosystems, marine-derived filamentous fungi represent an extraordinarily diverse biotic population. They distribute in almost all marine habitats, including marine plants, marine invertebrates and vertebrates, and marine sediments [2,6,7,8]. Among them, marine alga-associated fungi have drawn considerable attention because they can synthesize valuable secondary metabolites with potential pharmacological properties [6,7].

**Figure 1:**
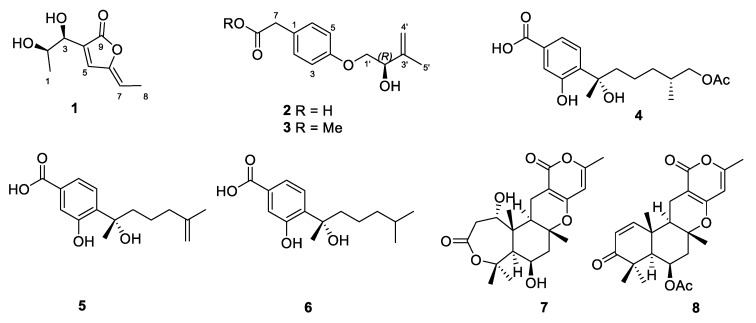
Chemical structures of **1**–**8**.

As part of our ongoing search for bioactive secondary metabolites from marine-derived fungi, the fungus *Penicillium chrysogenum* LD-201810 was isolated from the marine red alga *Grateloupia turuturu*. Subsequent chemical investigation of an EtOAc extract of the culture of this fungal strain led to the isolation of a new pentaketide derivative, penilactonol A (**1**), and two previously unreported hydroxyphenylacetic acid derivatives, (2’*R*)-stachyline B (**2**) and (2’*R*)-westerdijkin A (**3**), together with five known metabolites, bisabolane-type sesquiterpenoids **4**–**6** and meroterpenoids **7** and **8** (Figure 1). The structures and absolute configurations of the stereogenic carbons were unequivocally determined using extensive spectroscopic analyses, (Mo_2_(OAc)_4_)-induced circular dichroism (ICD), time-dependent density-functional theory (TDDFT) electronic circular dichroism (ECD) calculations, and single-crystal X-ray diffraction analyses. To the best of our knowledge, **2** and **3** adapt the 2’*R*-configuration as compared to known hydroxyphenylacetic acid-derived and *O*-prenylated natural products. Details of the isolation, structure elucidation, and biological activities of the isolated compounds are presented herein.

## 2. Results and Discussion

### 2.1. Structure Elucidation

**Figure 2:**
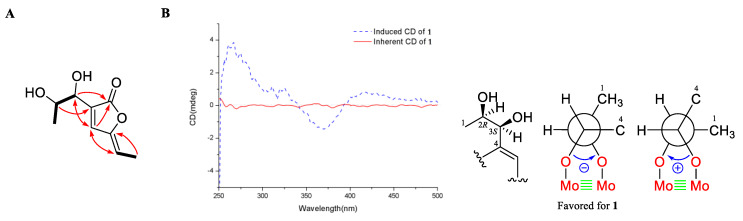
(**A**) COSY and key HMBC correlations in **1**; (**B**) induced circular dichroism (ICD) spectra from the Mo_2_-complex and inherent CD of **1**.

**Figure 3:**
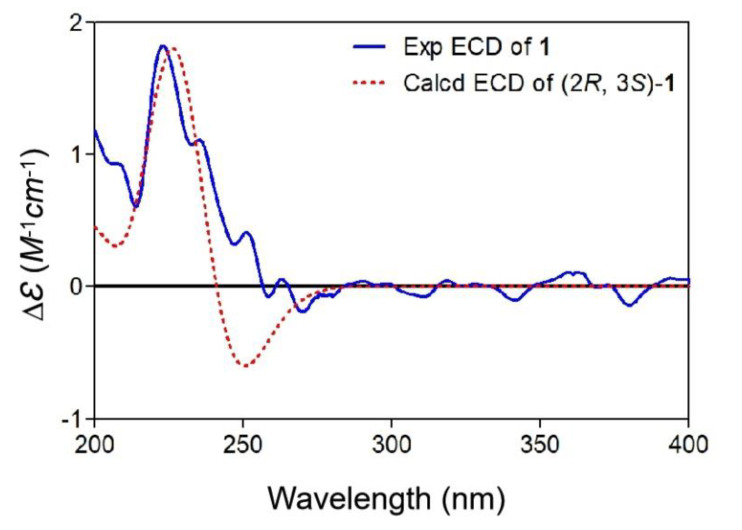
Experimental electronic circular dichroism (ECD) spectrum of **1** (blue solid); calculated ECD spectrum of (2*R*, 3*S*)-**1** (UV correction = −19 nm, red dash) at the CAM-B3LYP-SCRF/def2-SVP//B3LYP/6-31G(d) level of theory in MeOH with IEFPCM solvent model (Polarized Continuum Model using the Intergral Equation Formalism).

Compound **1** was obtained as a colorless oil with a molecular formula of C_9_H_12_O_4_, established by (+)-HRESIMS *m/z* 185.0803 [M + H]^+^, corresponding to four degrees of unsaturation. The ^1^H NMR spectrum (Table 1) showed two methyl doublets at *δ*_H_ 1.39 (d, *J* = 6.6 Hz, H_3_-1) and 1.88 (d, *J* = 7.4 Hz, H_3_-8), one doublet at *δ*_H_ 4.47 (d, *J* = 5.4 Hz, H-3), and a multiplet at *δ*_H_ 4.36 (m, H-2), and one singlet at *δ*_H_ 7.67 (s, H-5) and one quartet at *δ*_H_ 5.60 (q, *J* = 7.4 Hz, H-7) attributable to two olefinic protons. The ^13^C NMR spectrum, along with distortionless enhancement by polarization transfer (DEPT) and HSQC data, demonstrated the presence of two methyls at *δ*_C_ 19.7 (C-1) and 11.7 (C-8); four methines, including two oxygenated sp^3^ at *δ*_C_ 59.3 (C-2) and 69.1 (C-3) and two sp^2^ at *δ*_C_ 140.4 (C-5) and 112.2 (C-7); and three quaternary carbons, including two sp^2^ carbons at *δ*_C_ 132.1 (C-4) and 148.6 (C-6) and one carbonyl carbon at *δ*_C_ 168.4 (C-9). The COSY correlations (Figure 2A) from H_3_-1 to H-2 and from H-2 to H-3, combined with the downfield chemical shifts of C-2 (*δ*_C_ 59.3) and C-3 (*δ*_C_ 69.1), revealed a presence of a *vic*-diols moiety. The key HMBC correlations from H-2 to C-4, H-3 to C-5, H-5 to C-3 and C-7, H_3_-8 to C-6, and the COSY correlation of H-7 and H_3_-8 extended the fragment to C-4‒C-8. The HMBC correlations from H-3 and H-5 to C-9 located the carbonyl carbon C-9 linked to C-4. To satisfy the molecular formula and a degree of unsaturation, C-9 should connect to C-6 by an ester linkage to form the α,β-unsaturated γ-lactone ring. Hence, the planar structure of **1** was assigned. The key NOE correlation (Supplementary Materials, Figure S6) between H-5 and H-7 assigned the *Z* configuration of the double bond between C-6 and C-7. According to the literature, the coupling constant between the H-2 and H-3 is larger than 4 Hz in *erythro* isomers but smaller than 2 Hz in *threo* isomers in the α,β-unsaturated γ-lactones [9]. Therefore, the coupling constant of 5.4 Hz between H-2 and H-3 indicated the *erythro* relative configuration of 2,3-diol in **1** [10]. The absolute configuration of the *erythro*-2,3-diol in **1** was determined by the dimolybdenum-induced circular dichroism (ICD) analysis [11]. In the ICD analyses using Snatzke’s method with dimolybdenum tetraacetate [Mo_2_(OAc)_4_] in MeOH, the Mo_2_-complex of **1** gave a negative CD Cotton effect near 400 nm (Figure 2B). Using Snatzke’s helicity rule [11,12,13], the sign of the O–C–C–O torsional angle in the favored conformation of the chiral Mo_2_-complex determines the sign of the CD Cotton effect near 400 nm, and the conformation with an antiperiplanar orientation of the OH and the methyl group, O–C–C–CH_3_, is a favored conformation of the Mo_2_-complex in the *erythro*-diols closely resembling **1**, as shown in Figure 2B. Furthermore, the TDDFT method was employed at the CAM-B3LYP-SCRF/def2-SVP//B3LYP/6-31G(d) level to obtain the calculated ECD spectra of **1**. The experimental ECD spectrum of **1** was in good agreement with that of the calculated for (2*R*, 3*S*)-**1** at this level (Figure 3). Hence, the absolute configurations at C-2 and C-3 in **1** were finally determined to be 2*R*, 3*S*, respectively.

**Figure 4:**
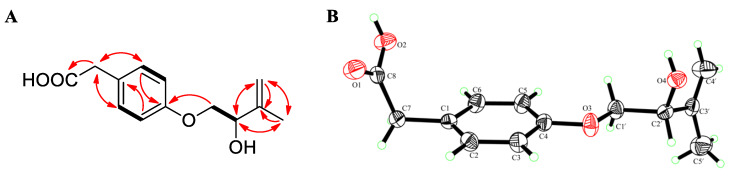
(**A**) COSY and key HMBC correlations of **2**; (**B**) ORTEP (Oak Ridge Thermal-Ellipsoid Plot Program) diagram for the single-crystal X-ray structure of **2**.

Compound **2** was acquired as colorless needles. The molecular formula C_13_H_16_O_4_ was established on the basis of (+)-HRESIMS data at *m/z* 254.1389 ([M + NH_4_]^+^) and 259.0942 ([M + Na]^+^). The ^1^H and ^13^C NMR data (Table 1), in combination with the HSQC spectrum, displayed signals that were attributed to one methyl at *δ*_H_ 1.72 (s, H-5’) and *δ*_C_ 18.9 (C-5’); three methylenes, including one oxygenated sp^3^ at *δ*_H_ 3.92 (dd, *J* = 9.9, 4.4 Hz, H-1’α), 3.84 (m, H-1’β), and *δ*_C_ 71.3 (C-1’), one sp^3^ at *δ*_H_ 3.46 (br s, H-7) and *δ*_C_ 40.2 (C-7), and one exocyclic sp^2^ at *δ*_H_ 4.86 (br s, H-4’α), 5.02 (br s, H-4’β) and *δ*_C_ 112.1 (C-4’); five methines, including four sp^2^ at *δ*_H_ 7.14 (d, *J* = 8.3 Hz, H-2/6), 6.86 (d, *J* = 8.4 Hz, H-3/5), *δ*_C_ 130.8 (C-2/6), 114.8 (C-3/5), and one sp^3^ at *δ*_H_ 4.24 (t, *J* = 5.3 Hz, H-2’) and *δ*_C_ 72.7 (C-2’); three non-protonated sp^2^ carbons at *δ*_C_ 127.4 (C-1), 157.8 (C-4), 145.8 (C-3’), and one carbonyl carbon at *δ*_C_ 173.4 (C-8). The above-mentioned spectroscopic features as well as the COSY correlation between H-2/6 and H-3/5 (Figure 4A) were interpreted as characteristic for a *para*-substituted aromatic ring. Besides, H_2_-1’ and H-2’ were coupled as evidenced by the COSY correlation (Figure 4A). The key HMBC correlations from H_3_-5’ to C-2’, C-3’, and C-4’, from H_2_-1’ to C-3’, and from H-2’ to C-4’ and C-5’ delineated an unsaturated and hydroxylated isoprene unit, (2-hydroxy-3-methylbut-3-en-1-yl)oxy (Figure 4A). Additional HMBC correlation from H_2_-1’ to C-4 showed that the isoprene unit was connected to the *para*-substituted aromatic ring via an oxygen atom. Furthermore, the interactive HMBC correlations from H_2_-7 to C-2, C-6, and C-8 constructed the other group (C7‒C8) linked to the aromatic ring. The planar structure of **2** was therefore determined. A literature search revealed that it possessed the same planar structure as stachyline B, a secondary metabolite previously isolated from the sponge-derived fungus *Stachylidium* sp. [14]. The configuration at C-2′ of stachyline B was determined as *S* by Mosher’s method [14]. Since **2** possessed the opposite sign of the optical rotation when compared to that of stachyline B (+16.1 in **2** vs −12 in stachyline B), the *R* configuration was proposed for C-2′ of **2**. X-ray diffraction crystallographic analysis enabled us to undoubtedly confirm its absolute configuration (Figure 4B).

Compound **3** was obtained as a white amorphous powder and possessed a molecular formula of C_14_H_18_O_4_ by (+)-HRESIMS data *m/z* 268.1548 [M + NH_4_]^+^ and 273.1095 [M + Na]^+^. With compound **2** in hand, the structure elucidation of **3** was quite straightforward. The ^1^H and ^13^C NMR spectra of **3** (Table 1) closely resembled those of **2**, except that **3** had one methyl group (*δ*_H/C_ 3.58/51.6, 8-OMe) more than **2**. Accordingly, **3** was elucidated as a methyl ester of **2**. Because **3** is dextrorotatory, it was concluded that **3** also has *R*-configuration at C-2’.

In addition, another five previously reported metabolites including bisabolane-type sesquiterpenoids **4**–**6** and meroterpenoids **7** and **8** were also isolated in this study. They were identified as (7*S*,11*S*)-(+)-12-acetoxysydonic acid (**4**) [15], (*S*)-(+)-11-dehydrosydonic acid (**5**) [15], sydonic acid (**6**) [16], asperdemin (**7**) [17], and asperversin G (**8**) [18], respectively, by comparing their NMR data with those from the literature.

### 2.2. Cytotoxicity of Compounds ***1***–***8***

**Figure 5:**
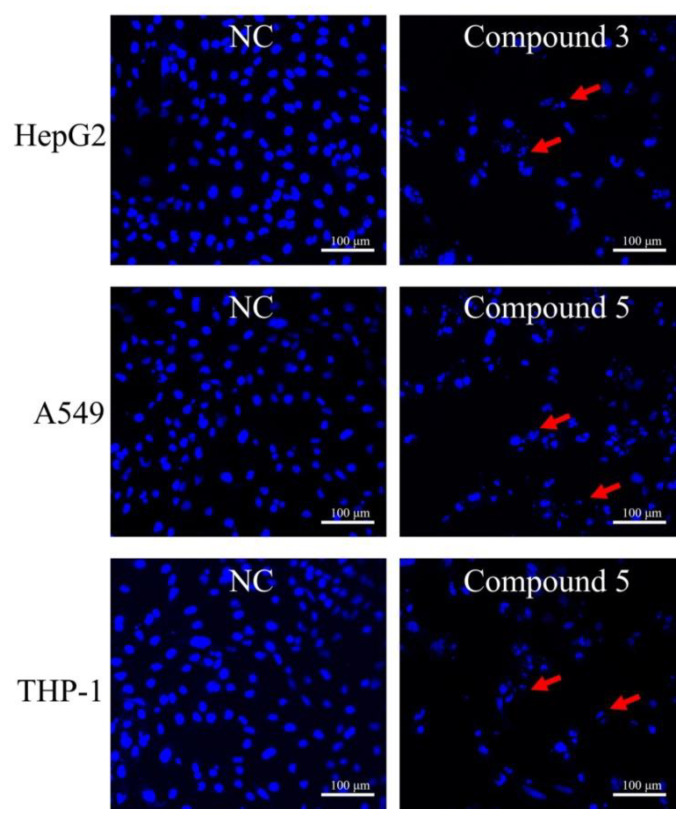
Apoptosis-related morphological changes were detected by staining cells with 4’,6-diamidino-2-phenylindole (DAPI). Apoptotic cells were defined as those with blue-stained cells that exhibited a fragmented/condensed nucleus and apoptotic body (red arrow).

The isolated compounds **1**–**8** were submitted to Cell Counting Kit-8 (CCK-8) colorimetric assays toward six human carcinoma cell lines (human lung adenocarcinoma epithelial cell line A549, human breast cancer cell line BT-549, human cervix carcinoma cell line HeLa, human liver carcinoma cell line HepG2, human breast adenocarcinoma cell line MCF-7, and human monocytic cell line THP-1) to estimate their cytotoxicities. Compound **3** exhibited cytotoxicity to the HepG2 cell line with an IC_50_ value of 22.0 μM. Furthermore, **5** also showed considerable activities against the A549 and THP-1 cell lines with IC_50_ values of 21.2 and 18.2 μM, respectively (Table 2). To determine whether the compounds could induce apoptosis, 4’,6-diamidino-2-phenylindole (DAPI) staining was conducted using a confocal laser scanning microscope. We found that many cells had typical apoptotic features, such as fragmented/condensed nucleus and apoptotic body formation (Figure 5). All staining results indicated that **3** and **5** had apoptosis-inducing activity against the HepG2, A549, and THP-1 cell lines, respectively.

## 3. Materials and Methods

### 3.1. General Experimental Procedures

The UV spectra were measured using a Shimadzu UV-2700 spectrometer (Shimadzu Co., Ltd., Kyoto, Japan). The optical rotations were measured using a Jasco P-1020 automatic polarimeter (JASCO, Tokyo, Japan). The NMR spectra were recorded on an Agilent DD2 500 MHz NMR spectrometer (Agilent Technologies, Waldbronn, Germany) with tetramethylsilane (TMS) as an internal standard. The HRESIMS data were obtained in the positive ion mode on a Waters Xevo G2-XS QTof mass spectrometer (Waters, Milford, MA, USA). Commercially available silica gel (100–200 and 200–300 mesh, Qingdao Marine Chemical Inc., Qingdao, China), Lobar LiChroprep RP-18 (40‒60 μm, Merck, Darmstadt, Germany), and Sephadex LH-20 (Merck) were used for open column chromatography.

### 3.2. Fungal Material

The fungal strain LD-201810 was previously isolated from the marine red alga *Grateloupia turuturu* collected in August 2016 from Qingdao, China. This strain was identified as *P*. *chrysogenum* according to its morphological characteristics and sequencing of the ITS region (GenBank no. MT075873). The strain was deposited in the Key Laboratory of Marine Biotechnology at the Universities of Shandong (Ludong University), School of Life Sciences, Ludong University, Yantai, China.

### 3.3. Fermentation, Extraction, and Isolation

The fermentation was performed statically on sterilized solid rice medium (70 g of rice, 0.1 g of corn flour, 0.3 g of peptone, 0.1 g of monosodium glutamate, and 100 mL of filtered seawater in each 1 L Erlenmeyer flask) at room temperature. After incubation for 30 days, a total of 50 flasks of cultured mycelium were exhaustively extracted with EtOAc (3 × 10 L). Then, the organic phase was filtered and evaporated under reduced pressure to afford 40.6 g of EtOAc crude extract. Chromatographic fractionation of the EtOAc crude extract was performed using an open silica gel vacuum liquid chromatography (VLC) gradient system (bed 10 × 60 cm, silica gel 300 g, 100–200 mesh); the column was eluted with mixtures of petroleum ether (PE)−EtOAc (from 5:1 to 1:1, *v*/*v*, collected 1 L for each fraction) and dichloromethane (DCM)−methanol (MeOH) (from 20:1 to 5:1, *v*/*v*, collected 1 L for each fraction). A total of eight fractions, i.e., fractions A (11.2 g), B (2.6 g), C (5.8 g), D (5.2 g), E (2.6 g), F (6.0 g), G (2.1 g), and H (1.9 g), were obtained and concentrated under reduced pressure. Fraction A (11.2 g), eluted with PE−EtOAc (5:1, *v*/*v*, collected 2 L eluent), was re-fractionated by a silica gel column (bed 8 × 80 cm, silica gel 200 g, 200–300 mesh; PE−EtOAc gradient, from 10:1 to 1:1, *v*/*v*) to yield subfractions A1 and A2. Fraction A1 (4.5 g, eluted with PE−EtOAc 5:1, *v*/*v*, collected 1 L eluent) was subjected to a Sephadex LH-20 column (MeOH, bed 1.5 × 135 cm) to obtain **7** (23.6 mg). Fraction A2 (3.2 g, eluted with PE−EtOAc 1:1, *v*/*v*, collected 1 L eluent) was purified using preparative thin layer chromatography (PTLC, plate: 20 × 20 cm; developing solvents: DCM−MeOH, 20:1, *v*/*v*, 160 mL) to obtain **8** (42.3 mg). Fraction C (5.8 g), eluted with PE−EtOAc (1:1, *v*/*v*, collected 1 L eluent), was further re-fractionated by a silica gel column (bed 8 × 80 cm, silica gel 200 g, 200–300 mesh; DCM−MeOH, from 20:1 to 10:1, *v*/*v*) to yield subfractions C1 and C2. Fraction C1 (1.2 g, eluted with DCM−MeOH 20:1, *v*/*v*, collected 500 mL eluent) was purified by a Sephadex LH-20 column (MeOH, bed 1.5 × 135 cm) to obtain **1** (10.5 mg). Fraction E (2.6 g) was subjected to reversed-phase column chromatography (bed 2 × 5 cm) over Lobar LiChroprep RP-18 with a MeOH−H_2_O gradient system (from 1:9 to 10:0, *v*/*v*, collected 1.2 L for each fraction) to afford five subfractions (Fr.E1−E5). Fr.E1 (123 mg, eluted with MeOH−H_2_O 3:7, *v*/*v*) was purified using prep-HPLC (SunFire^®^ C18, 250 mm × 10 mm, 5 μm; mobile phase: 50% MeOH−H_2_O; flow rate: 2 mL/min; UV detection: 235 nm) to afford **2** (8.5 mg, *t*_R_ 12.6 min). Fr.E2 (89 mg, eluted with MeOH−H_2_O 2:3, *v*/*v*) was subjected to PTLC (plate: 20 × 20 cm; developing solvents: DCM−acetone−acetic acid, 10:1:0.05, *v*/*v*, 80 mL) to afford **6** (9.5 mg). Fr.E3 (120 mg, eluted with MeOH−H_2_O 1:1, *v*/*v*) was chromatographed on a prep-HPLC column (SunFire^®^ C18, 250 mm × 10 mm, 5 μm; mobile phase: 60% MeOH−H_2_O; flow rate: 2 mL/min; UV detection: 235 nm) to obtain **3** (12.3 mg, *t*_R_ 15.0 min). Fr.E4 (200 mg, eluted with MeOH−H_2_O 3:2, *v*/*v*) was subjected to PTLC (plate: 20 × 20 cm; developing solvents: DCM−MeOH−CH_3_CO_2_H, 10:1:0.05, *v*/*v*, 4 × 40 mL) to afford **4** (11.3 mg) and **5** (26.3 mg).

*Penilactonol A* (**1**): colorless oil; [α]^25^_D_ +7.3° (*c* 0.20, MeOH); UV (MeOH) *λ*_max_ (log *ε*) 207 (0.22) nm; ECD (0.20 mg/mL, MeOH) *λ*_max_ (Δ*ε*) 224 (+1.80) nm; ^1^H and ^13^C NMR data, Table 1; (+)-HRESIMS *m/z* 185.0803 [M + H]^+^ (calcd for C_9_H_13_O_4_, 185.0808).

*(2′R)-Stachyline B* (**2**): colorless needles; mp 188–190 °C; [α]^25^_D_ +16.1° (c 0.25, MeOH); UV (MeOH) *λ*_max_ (log *ε*) 203 (3.37), 228 (3.81), 278 (3.12), 285 (2.79) nm; ^1^H and ^13^C NMR data, Table 1; (+)-HRESIMS *m/z* 254.1389 [M + NH_4_]^+^ (calcd for C_13_H_20_NO_4_, 254.1387) and 259.0942 [M + Na]^+^ (calcd for C_13_H_16_O_4_Na, 259.0941).

*(2′R)-Westerdijkin A* (**3**): white amorphous powder; [α]^25^_D_ +70.3° (c 0.30, MeOH); UV (MeOH) *λ*_max_ (log *ε*) 201 (3.33), 226 (3.79), 277 (3.06), 283 (2.80) nm; ^1^H and ^13^C NMR data, Table 1; (+)-HRESIMS *m/z* 268.1548 [M + NH_4_]^+^ (calcd for C_14_H_22_NO_4_, 268.1543) and 273.1095 [M + Na]^+^ (calcd for C_14_H_18_O_4_Na, 273.1097).

### 3.4. Measurement of ICD Spectrum of ***1*** Using Mo_2_(OAc)_4_

The ICD spectrum was measured using spectroscopy-grade anhydrous MeOH. A mixture of the ligand (**1**) and Mo_2_(OAc)_4_ in MeOH in an approximate 1:2 molar ratio was subjected to ICD measurement. The first CD spectrum was recorded immediately after mixing, and its time evolution was monitored until stationary ICD was reached about 10 min after mixing. After the inherent CD data of the compound were subtracted, the ICD spectrum was normalized to a molar concentration of **1** and was presented as the Δ*ε*′ values. The observed signs of the Cotton effect near 400 nm in the ICD were correlated to the absolute configuration of the 1,2-diol moiety [11].

### 3.5. Computational Section

The conformational search was conducted via the conformer–rotamer ensemble sampling tool (CREST) [19,20]. Density-functional theory (DFT) calculations were carried out using the Gaussian 16 program [21]. The conformers within an energy window of 4 kcal/mol were optimized with DFT calculations at the B3LYP/6-31G(d) level of theory with Grimme’s D3 dispersion correction. Next, energies of all optimized conformations were evaluated by M06-2X/6-311+g(2d,p) with D3 dispersion correction. Those conformers accounting for over 98% of the population were subjected to TDDFT ECD calculations at the CAM-B3LYP/def2-SVP level of theory in MeOH with the IEFPCM solvent model, respectively. For each conformer, 30 excited states were calculated [22]. The calculated ECD curves were generated using Multiwfn 3.6 software with a full width at half maximum (FWHM) for each peak set to 0.4 eV [23].

### 3.6. X-ray Crystallographic Analysis of ***2***

Single-crystal X-ray diffraction data of **2** were obtained on an Agilent Xcalibur Eos Gemini Charge Couple Device (CCD) plate diffractometer using graphite monochromatized Cu/K*α* radiation (λ = 1.54178 Å). The structures were solved by direct methods with the SHELXTL software package [24]. All non-hydrogen atoms were refined anisotropically. The H atoms were located using geometrical calculations, and their positions and thermal parameters were fixed during the structure refinement. The structure was refined using full-matrix least-squares techniques [25]. Crystallographic data of **2** have been deposited in the Cambridge Crystallographic Data Centre (CCDC) with the CCDC number of 1999572. The data can be obtained free of charge via CCDC, 12 Union Road, Cambridge CB21EZ, UK (e-mail: deposit@ccdc.cam.ac.uk).

*Crystal data for compound***2**: C_13_H_16_O_4_, F.W. = 236.26, monoclinic space group P2(1), unit cell dimensions *a* = 5.8770(7) Å, *b* = 7.7029(13) Å, *c* = 27.763(4) Å, *V* = 1256.8(3) Å^3^, *α* = *β* = *γ* = 90°, *Z* = 4, *d*_calcd_ = 1.249 mg/m^3^, crystal dimensions 0.36 × 0.22 × 0.12 mm, *μ* = 0.762 mm^–1^, *F*(000) = 504. The 1774 measurements yielded 1500 independent reflections after equivalent data were averaged, and Lorentz and polarization corrections were applied. The final refinement gave *R*_1_ = 0.0379 and w*R*_2_ = 0.1050 [*I* > 2*σ*(*I*)]. The Flack parameter was −0.2(3) in the final refinement for all 1774 reflections with 157 Friedel pairs.

### 3.7. Cytotoxic Assays

The CCK-8 colorimetric method and DAPI staining were used to determine the cytotoxicities of compounds **1**−**8** against six human carcinoma cell lines (A549, BT-549, HeLa, HepG2, MCF-7, and THP-1) [26]. For the DAPI staining, HepG2, A549, and THP-1 cells were initially incubated for 24 h and then exposed to compounds for 48 h. Then, the cells were fixed with 70% ethanol. Subsequently, the cells were stained with 4 ng/mL DAPI at 4 °C for 5–10 min. Stained cells in each group were observed using a confocal laser scanning microscope.

### 3.8. Statistical Analysis

In this study, experiments were performed in triplicate and in parallel. For data analysis, the SPSS 21.0 software package (Chicago, IL, USA) was used to detect the half-maximal inhibitory concentration (IC_50_) value.

## 4. Conclusions

Secondary metabolites produced by marine-derived fungi have gained remarkable attention due to their intriguing structures and potential pharmacological applications. In this study, a new pentaketide derivative, penilactonol A (**1**), and two previously unreported hydroxyphenylacetic acid derivatives, (2’*R*)-stachyline B (**2**) and (2’*R*)-westerdijkin A (**3**), together with five known metabolites including bisabolane-type sesquiterpenoids **4**–**6** and meroterpenoids **7** and **8**, were isolated from the solid culture of marine alga-associated fungus *P. chrysogenum* LD-201810. It should be pointed out that **2** and **3** adapt the 2’*R*-configuration as compared to known hydroxyphenylacetic acid-derived and *O*-prenylated natural products. The cytotoxicities of the isolated compounds were evaluated. Compound **3** exhibited cytotoxicity to the HepG2 cell line with an IC_50_ value of 22.0 μM, whereas **5** showed considerable activities against A549 and THP-1 cell lines with IC_50_ values of 21.2 and 18.2 μM, respectively. Moreover, DAPI staining indicated that **3** and **5** had apoptosis-inducing activity. The present study may provide further proof that marine natural products are promising candidates for the discovery of new lead compounds of antitumor drugs.

## Figures and Tables

**Table 1:** ^1^H (500 MHz) and ^13^C NMR (125 MHz) data of compounds **1**‒**3** in DMSO-*d*_6_.

No.	Compound 1		No.	Compound 2		Compound 3	
	*δ*_H_ (Mult, *J* in Hz)	*δ*_C_, Type		*δ*_H_ (Mult, *J* in Hz)	*δ*_C_, Type	*δ*_H_ (Mult, *J* in Hz)	*δ*_C_, Type
1	1.39 (d, 6.6)	19.7, CH_3_	1		127.4, C		126.3, C
2	4.36 (m)	59.3, CH	2/6	7.14 (d, 8.3)	130.8, CH	7.15 (d, 8.6)	130.4, CH
3	4.47 (d, 5.4)	69.1, CH	3/5	6.86 (d, 8.3)	114.8, CH	6.88 (d, 8.6)	114.5, CH
4		132.1, C	4		157.8, C		157.6, C
5	7.67 (s)	140.4, CH	7	3.46 (br s)	40.2, CH_2_	3.58 (d, 5.3)	39.3, CH_2_
6		148.6, C	8		173.4, C		171.9, C
7	5.60 (q, 7.4)	112.2, CH	1’	3.92 (dd, 9.9, 4.4) 3.84 (m)	71.3, CH_2_	3.93 (dd, 9.9, 4.5) 3.86 (dd, 9.9, 6.9)	70.9, CH_2_
8	1.88 (d, 7.4)	11.7, CH_3_	2’	4.24 (t, 5.3)	72.7, CH	4.25 (m)	72.3, CH
9		168.4, C	3’		145.8, C		145.4, C
			4’	4.86 (br s) 5.02 (br s)	112.1, CH_2_	4.87 (br s) 5.03 (br s)	111.7, CH_2_
			5’	1.72 (s)	18.9, CH_3_	1.73 (s)	18.5, CH_3_
			8-OMe			3.58 (s)	51.6, CH_3_
			2’-OH			5.23 (br s)	

**Table 2:** Cytotoxicity of **1**–**8** (IC_50_, μM, mean ± SD, n = 3).

Compound	A549	BT-549	HeLa	HepG2	MCF-7	THP-1
**1**	>100	>100	>100	>100	>100	>100
**2**	>100	87.3 ± 3.5	96.6 ± 1.5	>100	>100	>100
**3**	70.0 ± 1.2	>100	>100	22.0 ± 1.2	>100	>100
**4**	63.6 ± 2.6	>100	78.7 ± 2.9	>100	>100	78.7 ± 1.9
**5**	21.2 ± 2.3	>100	61.7 ± 2.2	>100	>100	18.2 ± 1.2
**6**	>100	>100	>100	>100	>100	>100
**7**	>100	>100	>100	>100	>100	>100
**8**	>100	>100	>100	>100	>100	>100
Epirubicin ^a^	6.6 ± 0.5	4.4 ± 0.2	2.2 ± 0.3	3.7 ± 0.2	3.9 ± 0.1	4.8 ± 0.2

^a^ Positive control.

## Supplementary Materials

The following are available online at https://www.mdpi.com/1660-3397/18/5/276/s1. Figures S1–S19: ^1^H NMR, ^13^C NMR, HSQC, ^1^H-^1^H COSY, HMBC, NOESY, and HRESIMS spectra of compounds **1**−**3**; Figures S20–S28: Cytotoxicity of **1**–**8** and epirubicin; Tables S1–S19: Gibbs free energies and equilibrium populations of the calculated conformers.
