# Supplementary material for: Cytotoxic Secondary Metabolites Isolated from the Marine Alga-Associated Fungus Penicillium chrysogenum LD-201810
Source: Mar Drugs. 2020 May 22;18(5):276. doi: 10.3390/md18050276 (PMC7281594; doi:10.3390/md18050276)
Supplement: Supplementary file 1 [file marinedrugs-18-00276-s001.pdf]

Supplementary Materials

Cytotoxic Secondary Metabolites Isolated from the Marine Alga-Associated Fungus *Penicillium chrysogenum* LD-201810

Lin-Lin Jiang [1,2,3,†], Jin-Xiu Tang [1,†], Yong-Heng Bo [2], You-Zhi Li [2], Tao Feng [2], Hong-Wei Zhu [1,2,3], Xin Yu [1,2,3], Xing-Xiao Zhang [1,3*], Jian-Long Zhang [1,2,3*], and Weiyi Wang [4*]

1 School of Life Sciences, Ludong University, Yantai 264025, China; linlinjiang1986@163.com (L.-L. J.); 2948199639@qq.com (J.-X. T.); hngwzhu@outlook.com (H.-W. Z.); yuxinzghn@163.com (X. Y.)

2 Shandong Provincial Key Laboratory of Quality Safty Monitoring and Risk Assessment for Animal Products, Ji'nan 250022, China; yongheng1980@163.com (Y.-H. B.); Liyouzhi2009@qq.com (Y.-Z. L.); 41753392@qq.com (T. F.)

3 Yantai Key Laboratory of Animal Pathogenetic Microbiology and Immunology, Yantai 264025, China

4 Key Laboratory of Marine Biogenetic Resources, Third Institute of Oceanography, Ministry of Natural Resources, Xiamen 361005, China

* Correspondence: zhangjianlong@ldu.edu.cn (J.-L. Z.); zhangxingxiao@ldu.edu.cn (X.-X. Z.); wywang@tio.org.cn (W. W.); Tel.: +86-535-6681162 (J.-L. Z.); +86-535-6673485 (X.-X. Z.); +86-592-219-5518 (W. W.)

† These authors contributed equally to this work.

Table of Contents

Figure S1. ^{1}H NMR (500 MHz, DMSO-d_6) spectrum of compound **1**;

Figure S2. ^{13}C NMR (125 MHz, DMSO-d_6) and DEPT spectra of compound **1**;

Figure S3. HSQC spectrum of compound **1**;

Figure S4. COSY spectrum of compound **1**;

Figure S5. HMBC spectrum of compound **1**;

Figure S6. NOESY spectrum of compound **1**;

Figure S7. HRESIMS spectrum of compound **1**;

Figure S8. ^{1}H NMR (500 MHz, DMSO-d_6) spectrum of compound **2**;

Figure S9. ^{13}C NMR (125 MHz, DMSO-d_6) and DEPT spectra of compound **2**;

Figure S10. HSQC spectrum of compound **2**;

Figure S11. COSY spectrum of compound **2**;

Figure S12. HMBC spectrum of compound **2**;

Figure S13. HRESIMS spectrum of compound **2**;

Figure S14. ^{1}H NMR (500 MHz, DMSO-d_6) spectrum of compound **3**;

Figure S15. ^{13}C NMR (125 MHz, DMSO-d_6) and DEPT spectra of compound **3**;

Figure S16. HSQC spectrum of compound **3**;

Figure S17. COSY spectrum of compound **3**;

Figure S18. HMBC spectrum of compound **3**;

Figure S19. HRESIMS spectrum of compound **3**

Figure S20. Cytotoxicity of compound **1**

Figure S21. Cytotoxicity of compound **2**

Figure S22. Cytotoxicity of compound **3**

Figure S23. Cytotoxicity of compound **4**

Figure S24. Cytotoxicity of compound **5**

Figure S25. Cytotoxicity of compound **6**

Figure S26. Cytotoxicity of compound **7**

Figure S27. Cytotoxicity of compound **8**

Figure S28. Cytotoxicity of epirubicin

Table S1. Conformational analysis of the B3LYP/6-31G(d) optimized conformers of **1** in the gas phase (T=298.15 K)

Table S2. Atomic coordinates (Å) of **1**-1 obtained at the B3LYP/6-31G(d) level of theory in the gas phase.

Table S3. Atomic coordinates (Å) of **1**-2 obtained at the B3LYP/6-31G(d) level of theory in the gas phase.

Table S4. Atomic coordinates (Å) of **1**-3 obtained at the B3LYP/6-31G(d) level of theory in the gas phase.

Table S5. Atomic coordinates (Å) of **1**-4 obtained at the B3LYP/6-31G(d) level of theory in the gas phase.

Table S6. Atomic coordinates (Å) of **1**-5 obtained at the B3LYP/6-31G(d) level of theory in the gas phase.

Table S7. Atomic coordinates (Å) of **1**-6 obtained at the B3LYP/6-31G(d) level of theory in the gas phase.

Table S8. Atomic coordinates (Å) of **1**-7 obtained at the B3LYP/6-31G(d) level of theory in the gas phase.

Table S9. Atomic coordinates (Å) of **1**-8 obtained at the B3LYP/6-31G(d) level of theory in the gas phase.

Table S10. Atomic coordinates (Å) of **1**-9 obtained at the B3LYP/6-31G(d) level of theory in the gas phase.

Table S11. Key transitions, oscillator strengths, and rotatory strengths in the ECD spectrum of conformer **1**-1 at the CAM-B3LYP-SCRF/def2-SVP//B3LYP/6-31G(d) level of theory in MeOH with IEFPCM solvent model.

Table S12. Key transitions, oscillator strengths, and rotatory strengths in the ECD spectrum of conformer **1**-2 at the CAM-B3LYP-SCRF/def2-SVP//B3LYP/6-31G(d) level of theory in MeOH with IEFPCM solvent model.

Table S13. Key transitions, oscillator strengths, and rotatory strengths in the ECD spectrum of conformer **1**-3 at the CAM-B3LYP-SCRF/def2-SVP//B3LYP/6-31G(d) level of theory in MeOH with IEFPCM solvent model.

Table S14. Key transitions, oscillator strengths, and rotatory strengths in the ECD spectrum of conformer **1**-4 at the CAM-B3LYP-SCRF/def2-SVP//B3LYP/6-31G(d) level of theory in MeOH with IEFPCM solvent model.

Table S15. Key transitions, oscillator strengths, and rotatory strengths in the ECD spectrum of conformer **1**-5 at the CAM-B3LYP-SCRF/def2-SVP//B3LYP/6-31G(d) level of theory in MeOH with IEFPCM solvent model.

Table S16. Key transitions, oscillator strengths, and rotatory strengths in the ECD spectrum of conformer **1**-6 at the CAM-B3LYP-SCRF/def2-SVP//B3LYP/6-31G(d) level of theory in MeOH with IEFPCM solvent model.

Table S17. Key transitions, oscillator strengths, and rotatory strengths in the ECD spectrum of conformer **1**-7 at the CAM-B3LYP-SCRF/def2-SVP//B3LYP/6-31G(d) level of theory in MeOH with IEFPCM solvent model.

Table S18. Key transitions, oscillator strengths, and rotatory strengths in the ECD spectrum of conformer **1**-8 at the CAM-B3LYP-SCRF/def2-SVP//B3LYP/6-31G(d) level of theory in MeOH with IEFPCM solvent model.

Table S19. Key transitions, oscillator strengths, and rotatory strengths in the ECD spectrum of conformer **1**-9 at the CAM-B3LYP-SCRF/def2-SVP//B3LYP/6-31G(d) level of theory in MeOH with IEFPCM solvent model.

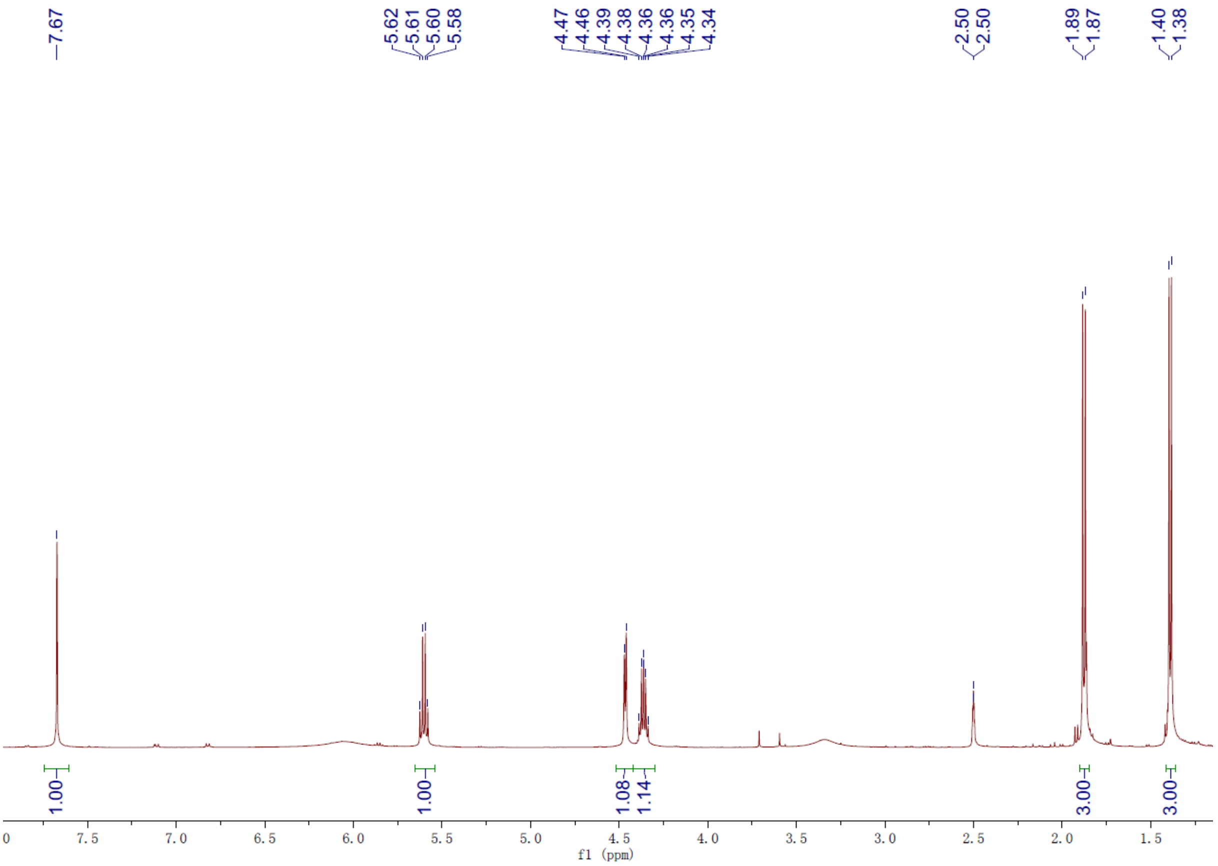

Figure S1. ^{1}H NMR (500 MHz, DMSO-d_6) spectrum of compound **1**;

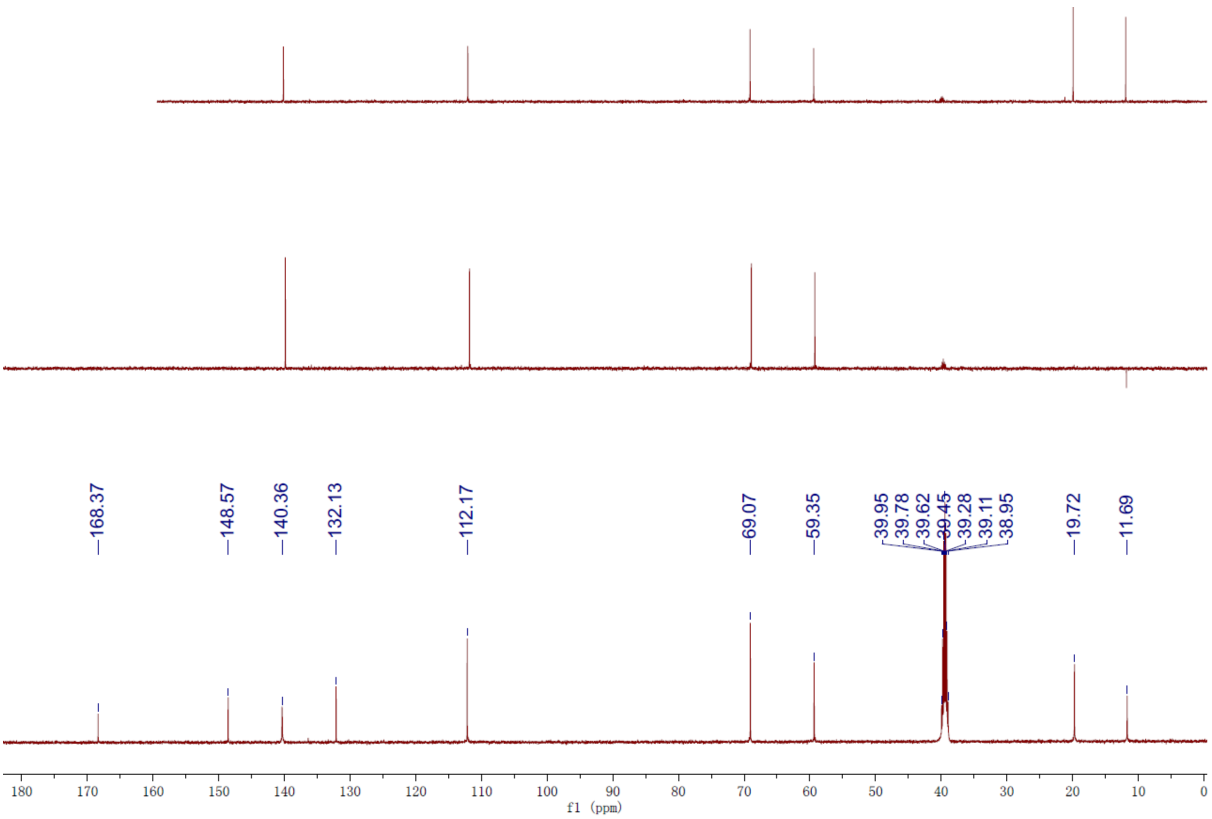

Figure S2. ^{13}C NMR (125 MHz, DMSO-d_6) and DEPT spectra of compound **1**;

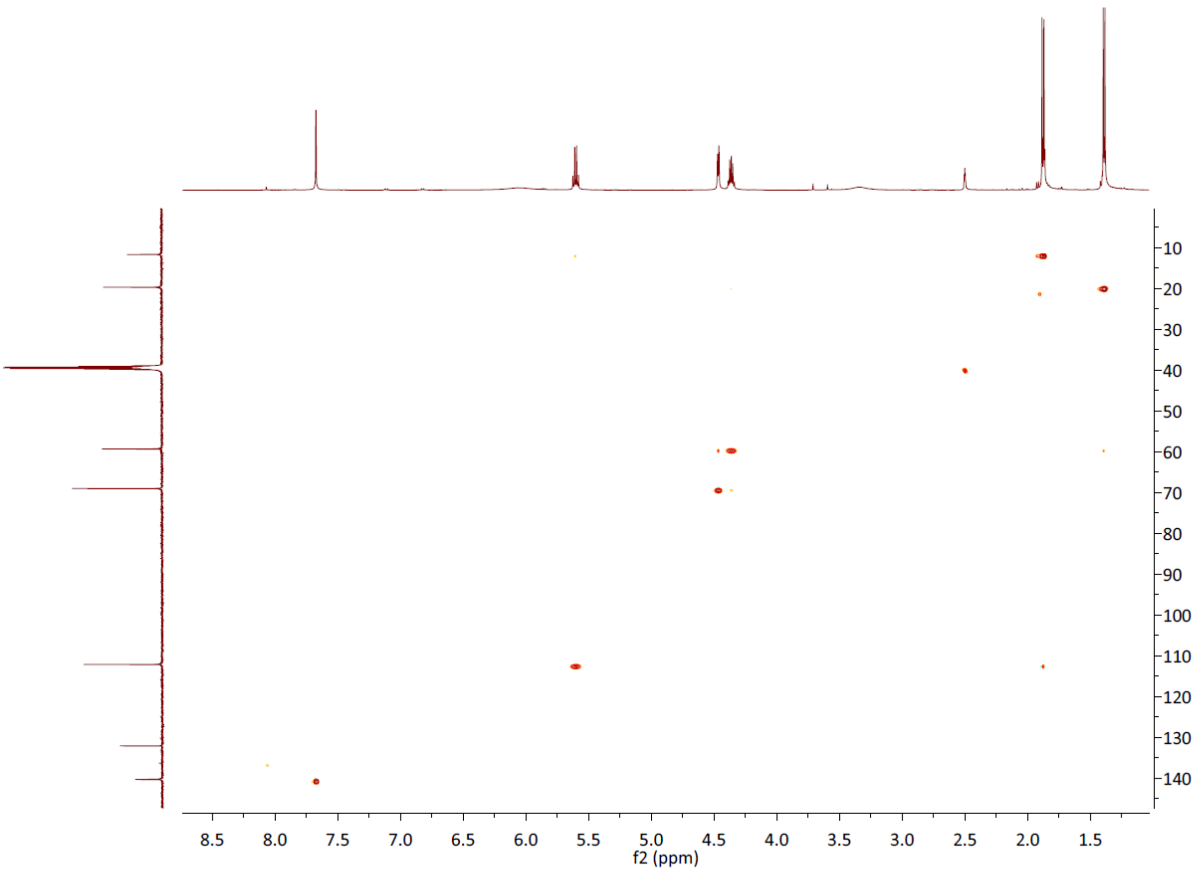

Figure S3. HSQC spectrum of compound **1**;

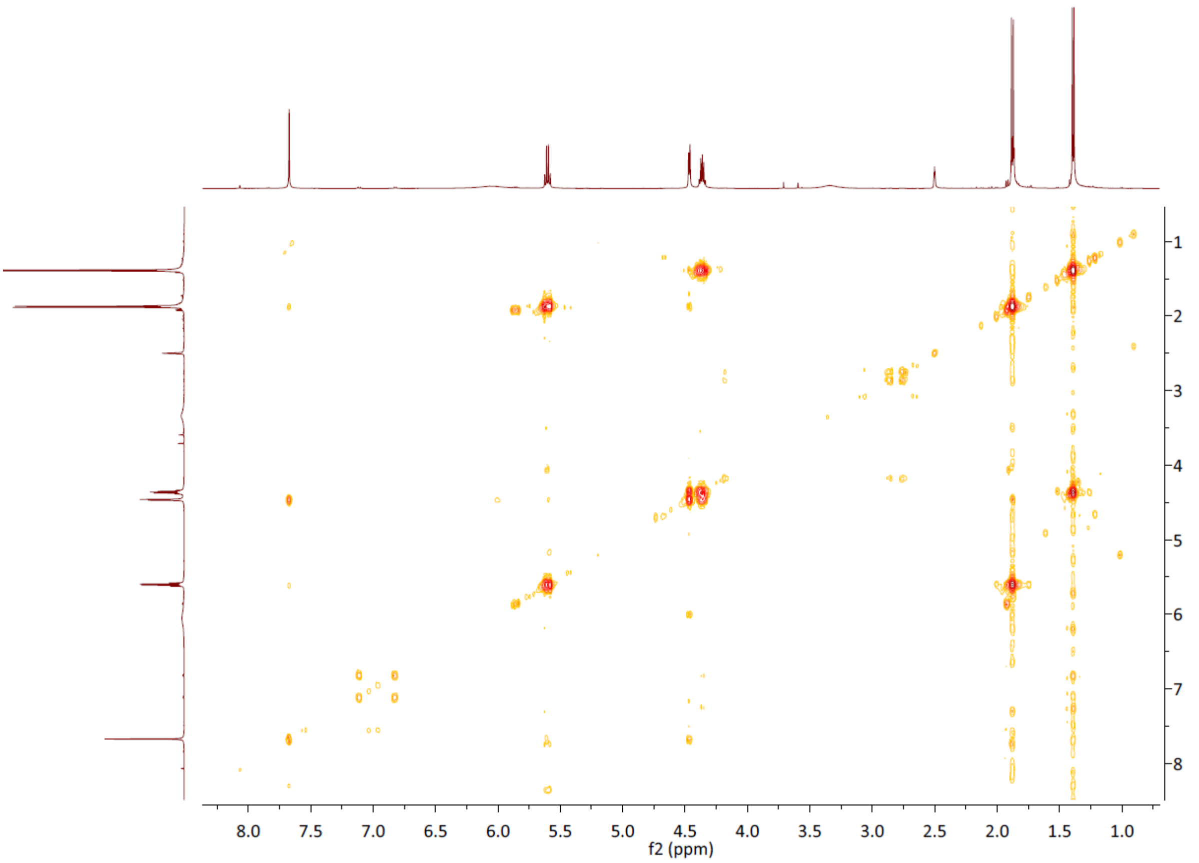

Figure S4. COSY spectrum of compound **1**;

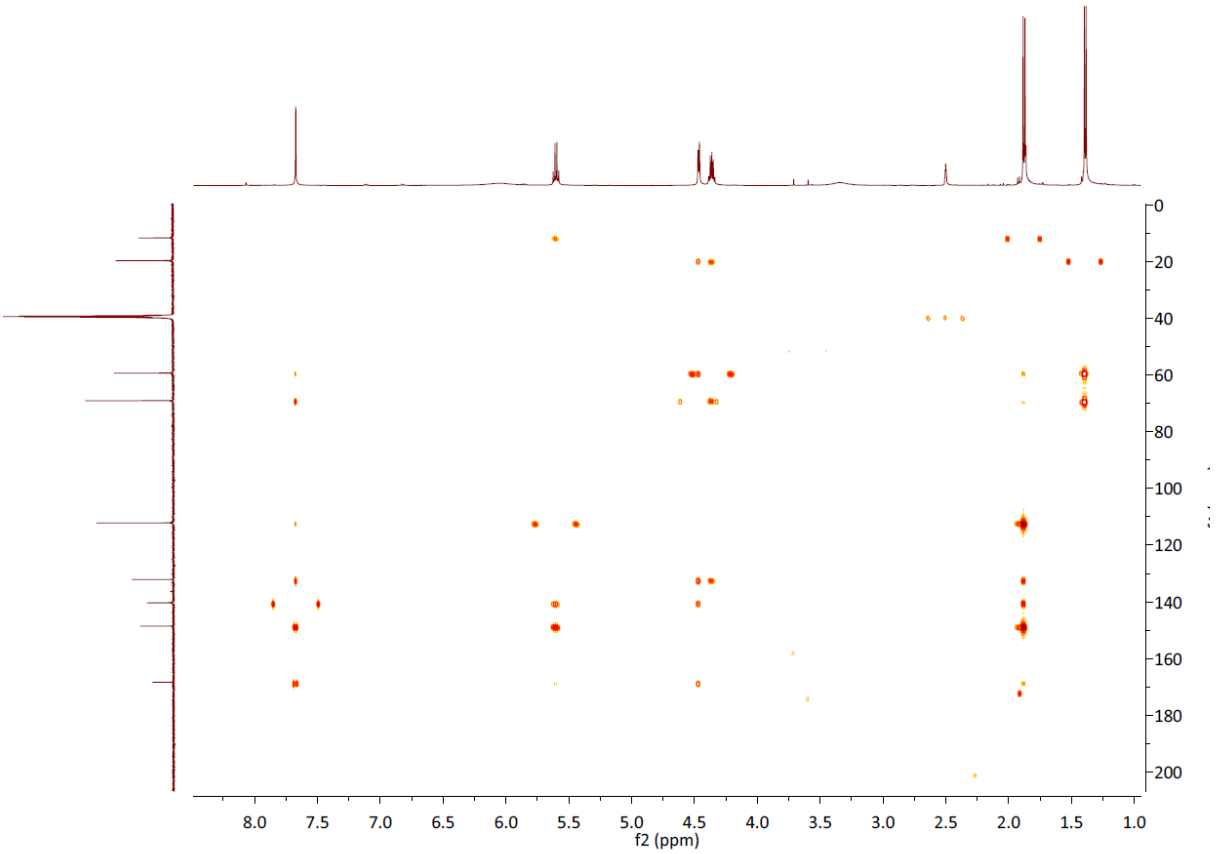

Figure S5. HMBC spectrum of compound **1**;

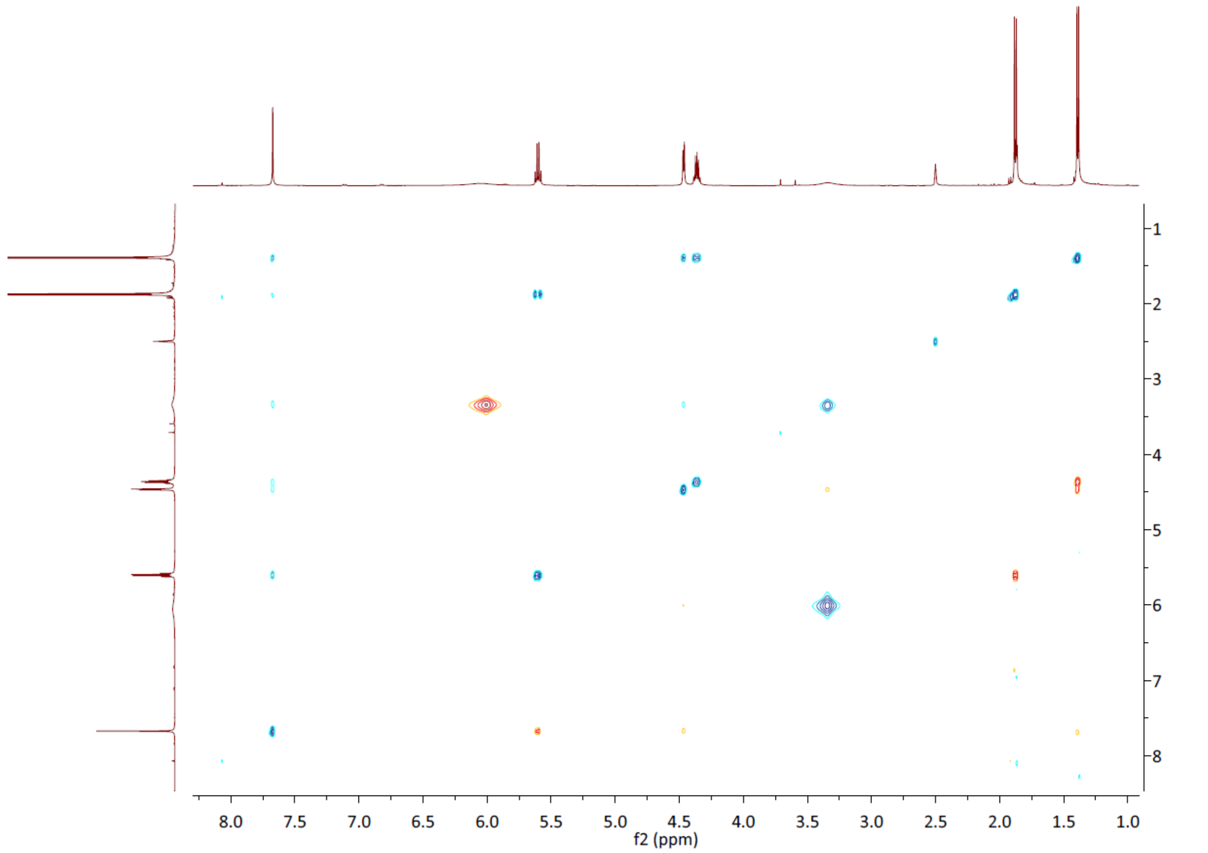

Figure S6. NOESY spectrum of compound **1**;

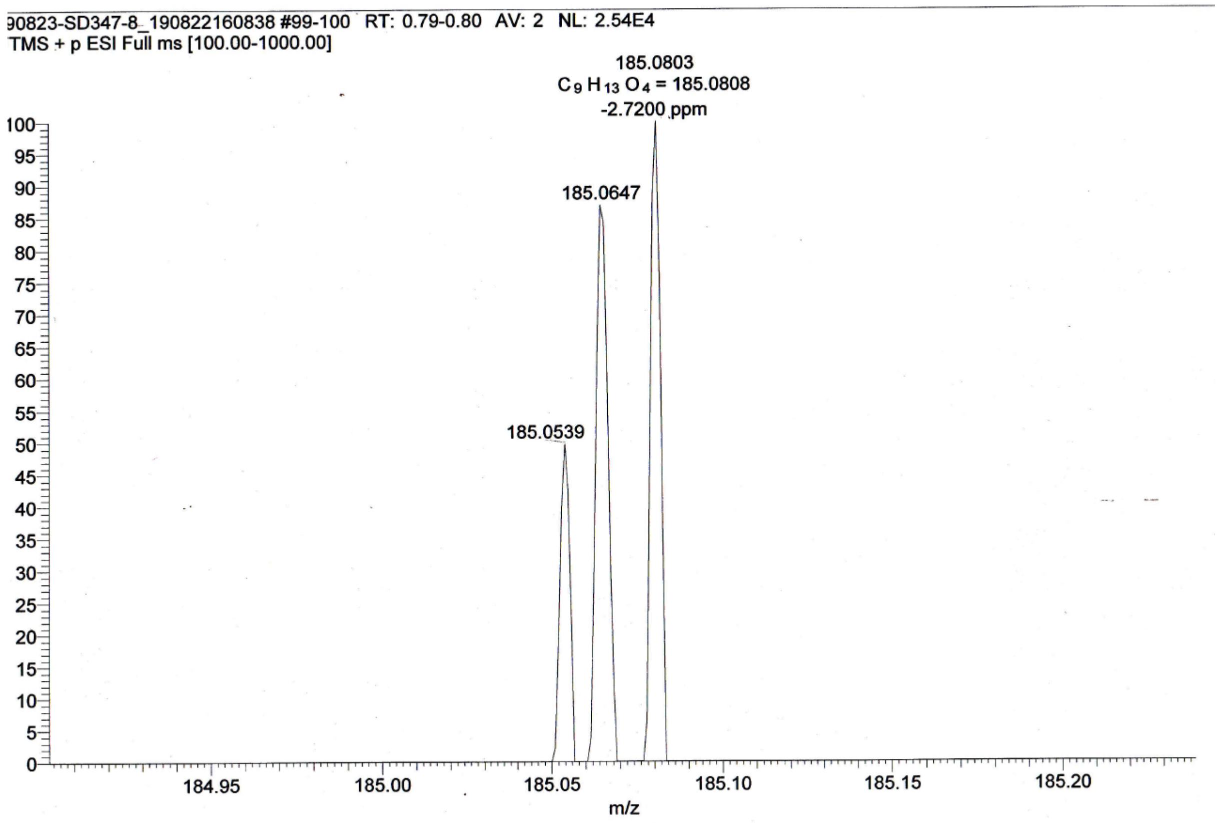

Figure S7. HRESIMS spectrum of compound **1**;

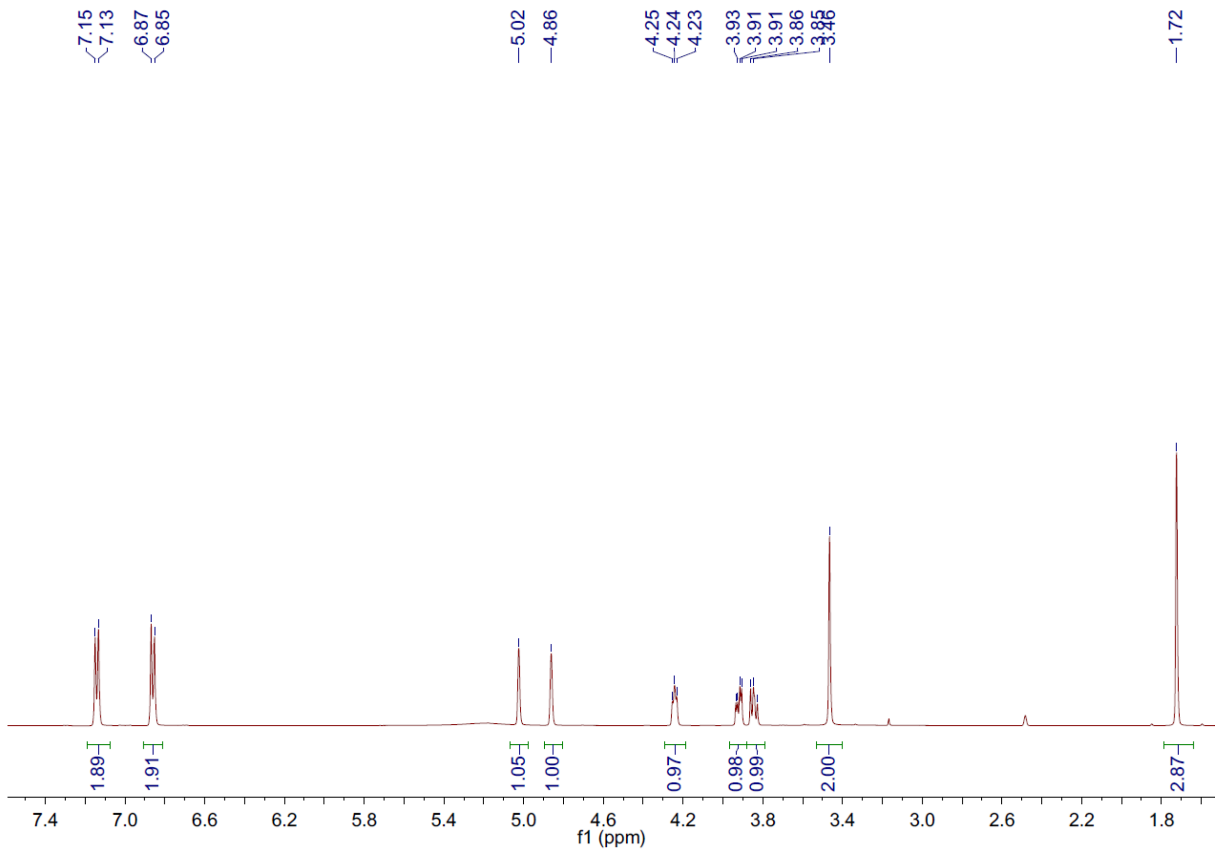

Figure S8. ^{1}H NMR (500 MHz, DMSO-d_6) spectrum of compound **2**;

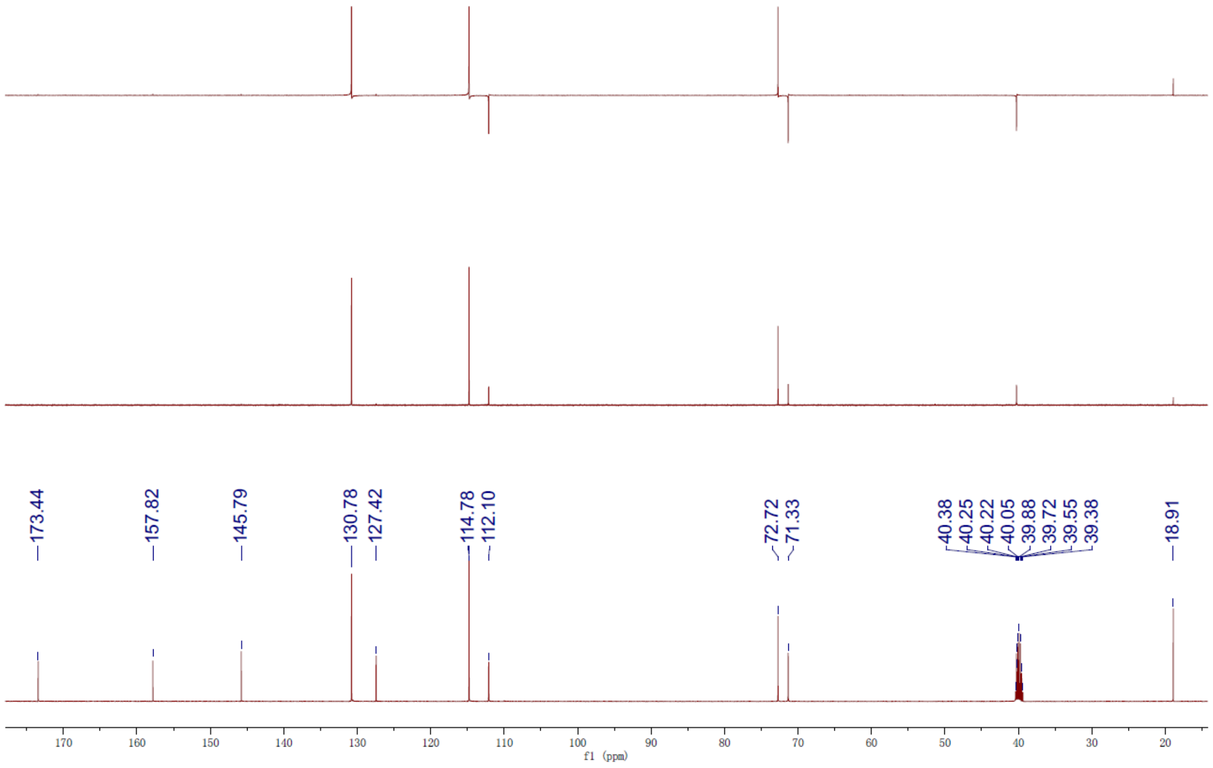

Figure S9. ^{13}C NMR (125 MHz, DMSO-d_6) and DEPT spectra of compound **2**;

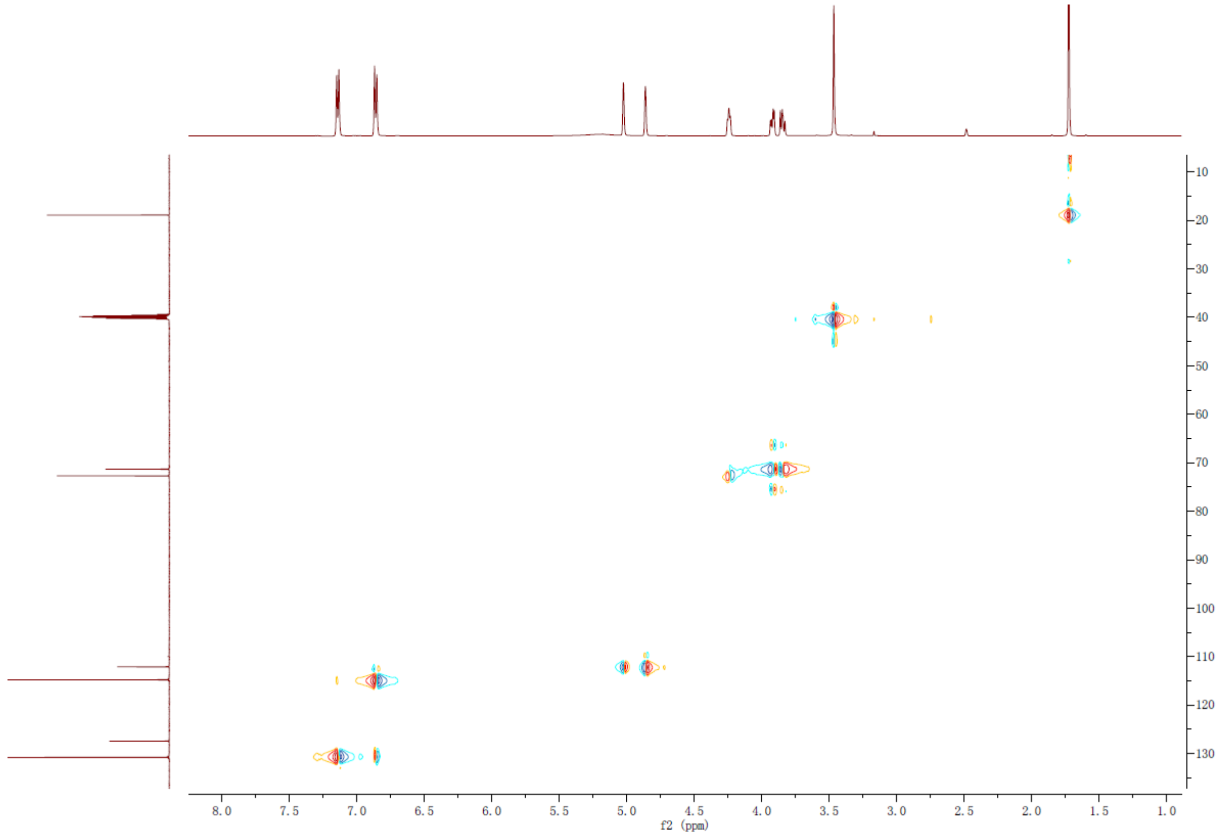

Figure S10. HSQC spectrum of compound **2**;

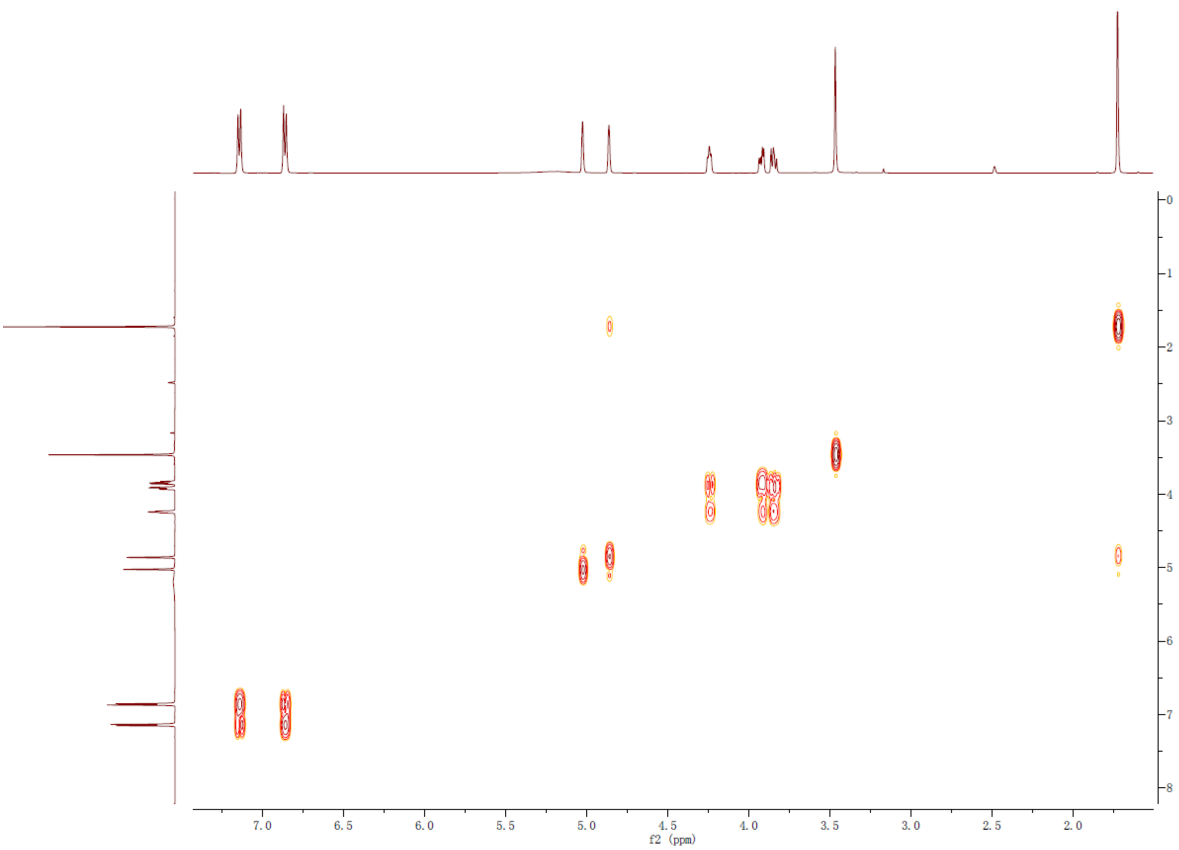

Figure S11. COSY spectrum of compound **2**;

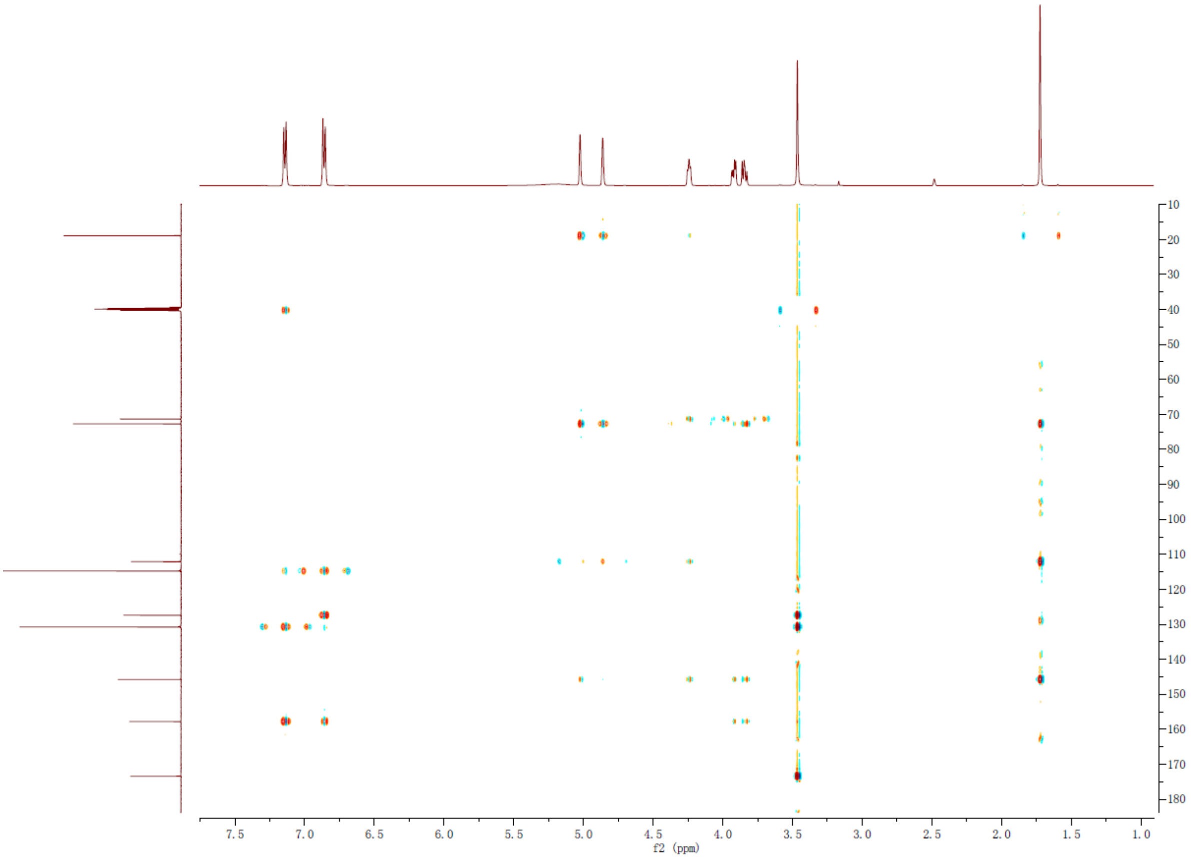

Figure S12. HMBC spectrum of compound **2**;

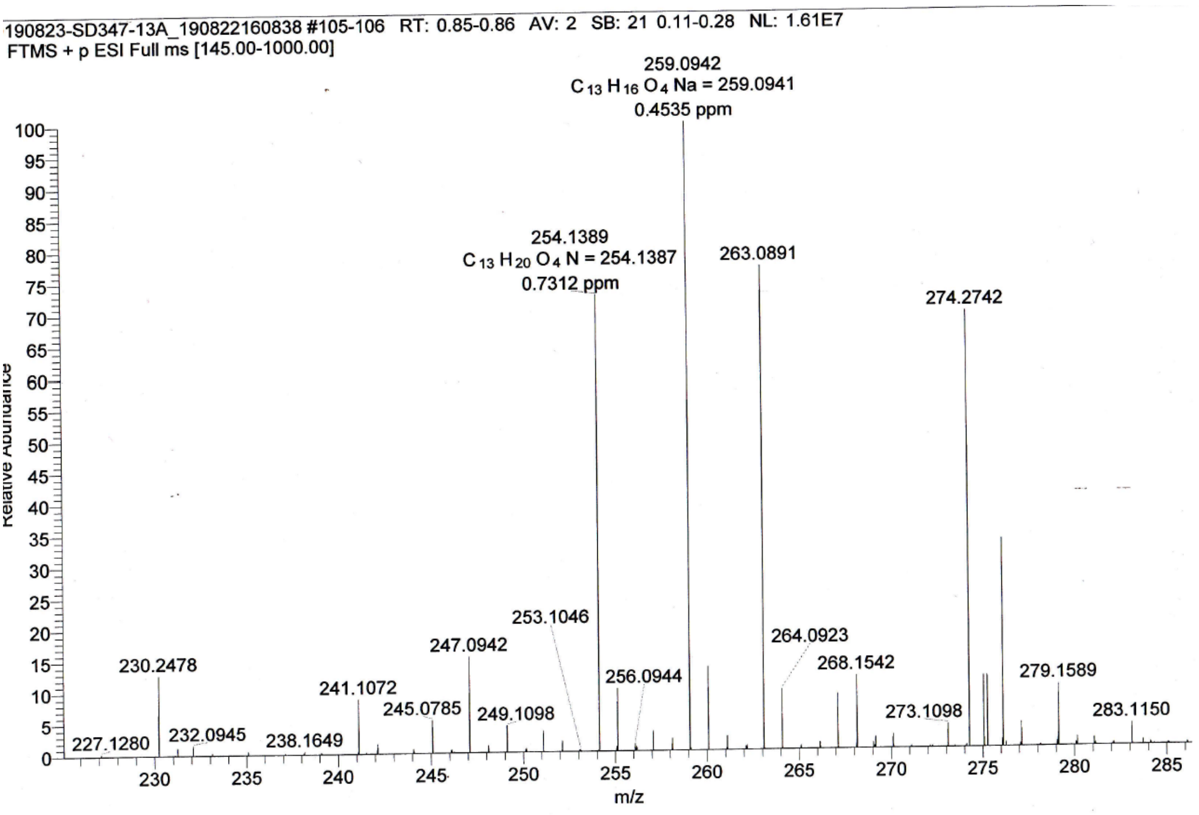

Figure S13. HRESIMS spectrum of compound **2**;

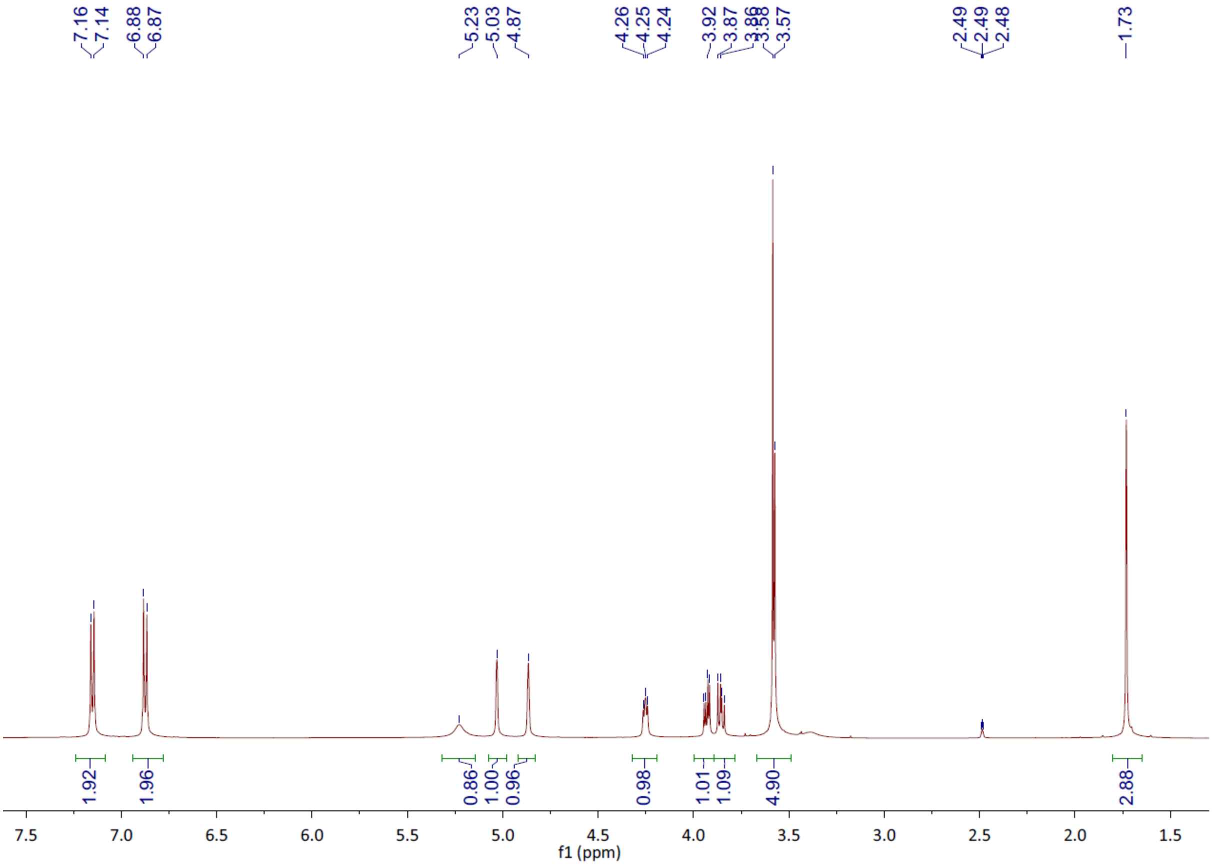

Figure S14. ^{1}H NMR (500 MHz, DMSO-*d*6) spectrum of compound **3**;

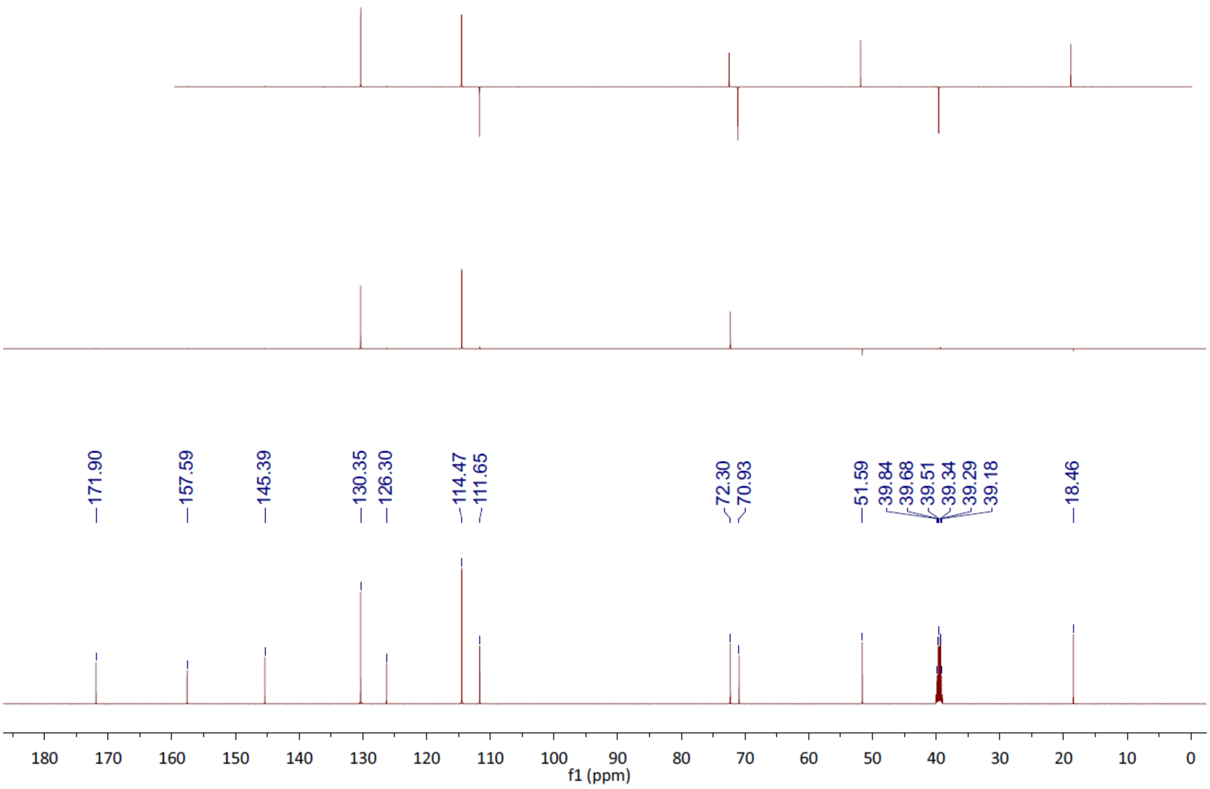

Figure S15. ^{13}C NMR (125 MHz, DMSO-d_6) and DEPT spectra of compound **3**;

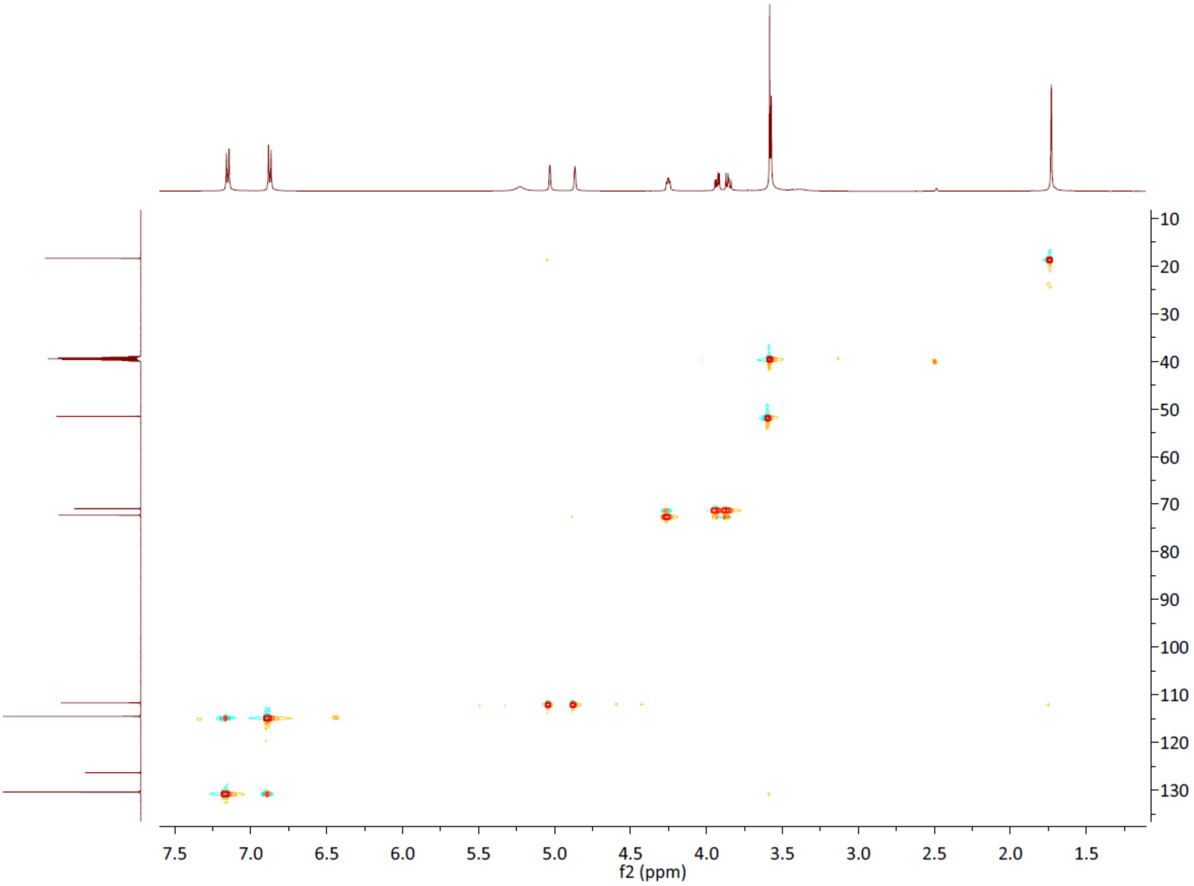

Figure S16. HSQC spectrum of compound **3**;

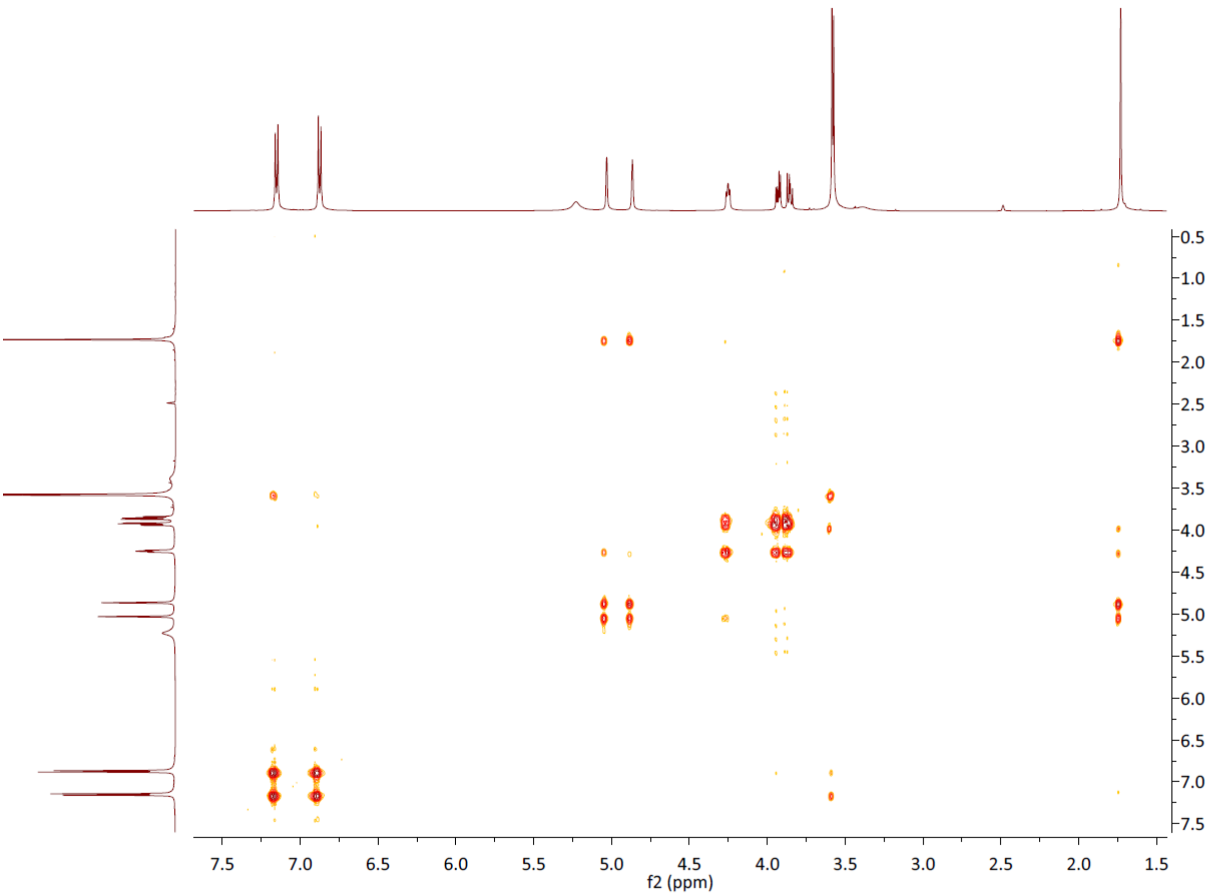

Figure S17. COSY spectrum of compound **3**;

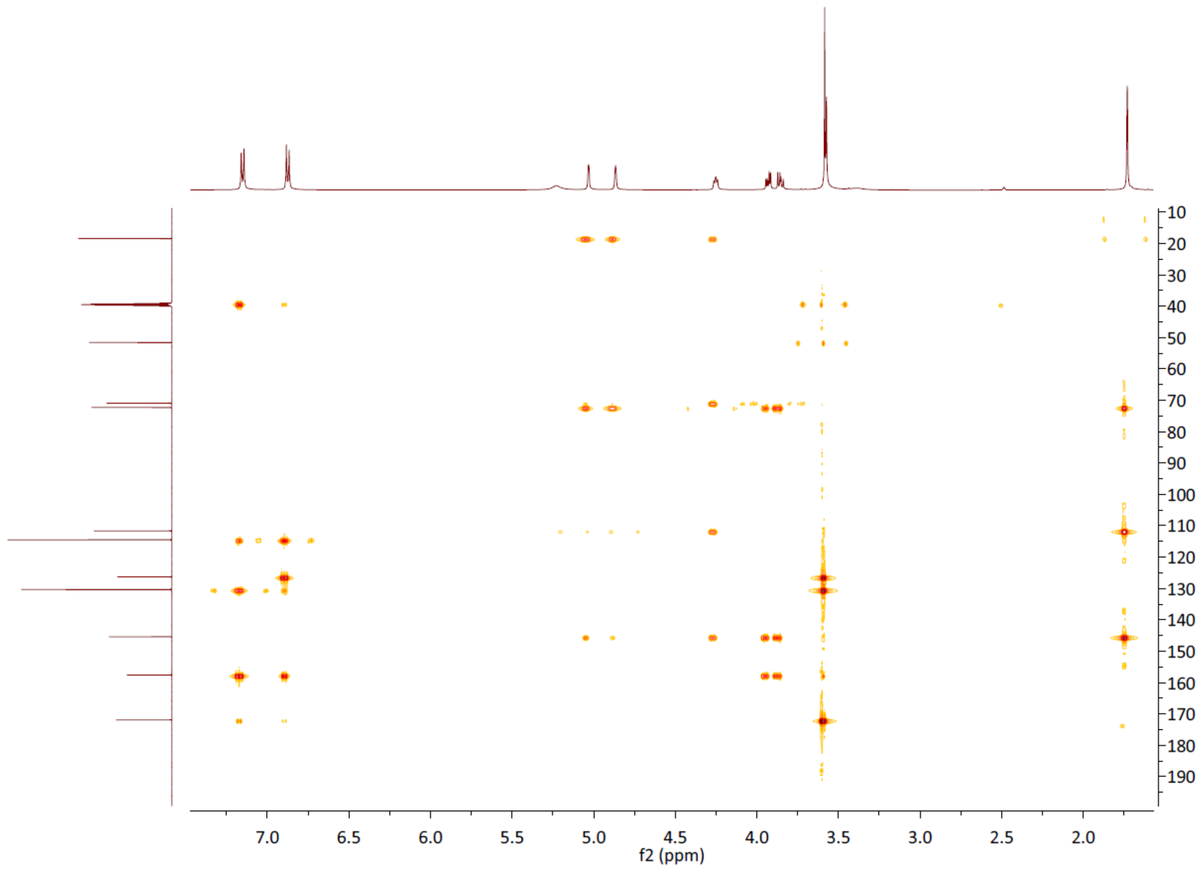

Figure S18. HMBC spectrum of compound **3**;

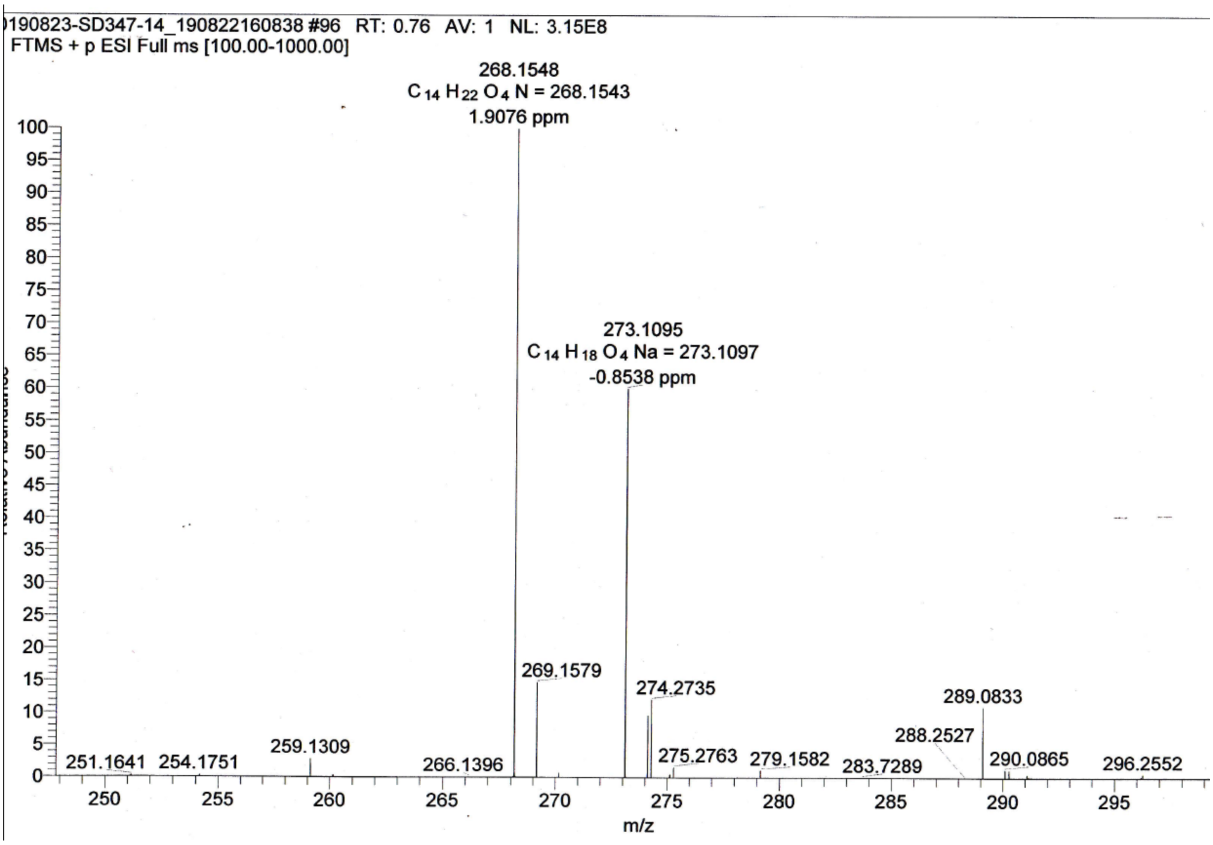

Figure S19. HRESIMS spectrum of compound **3**

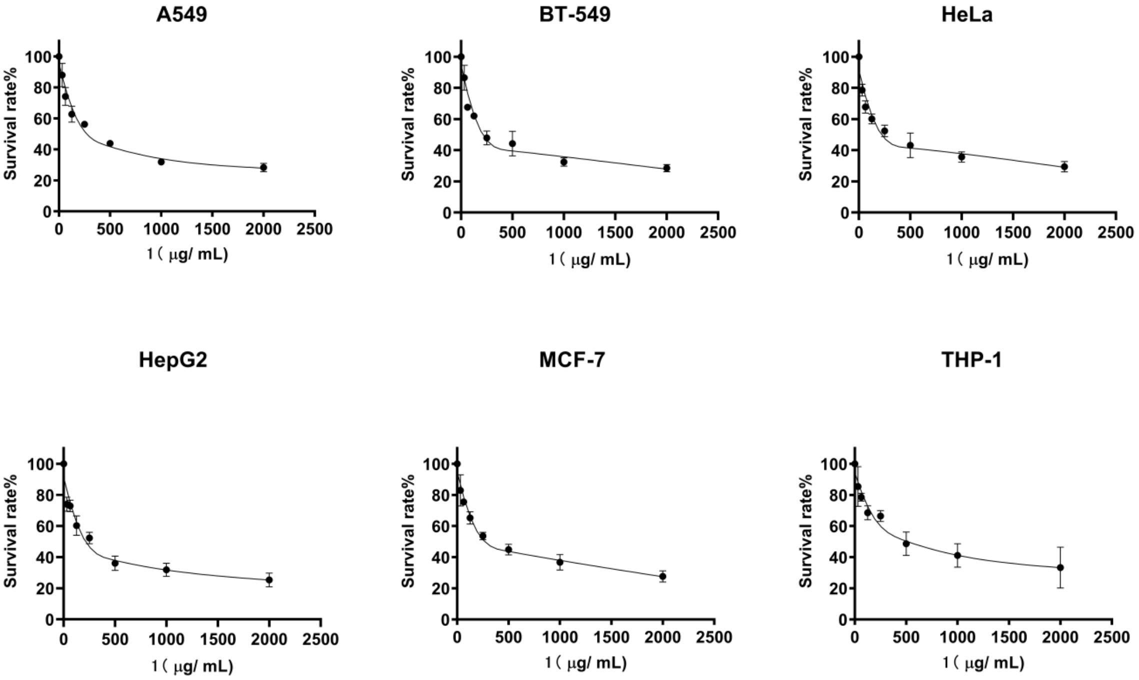

Figure S20. Cytotoxicity of compound **1**

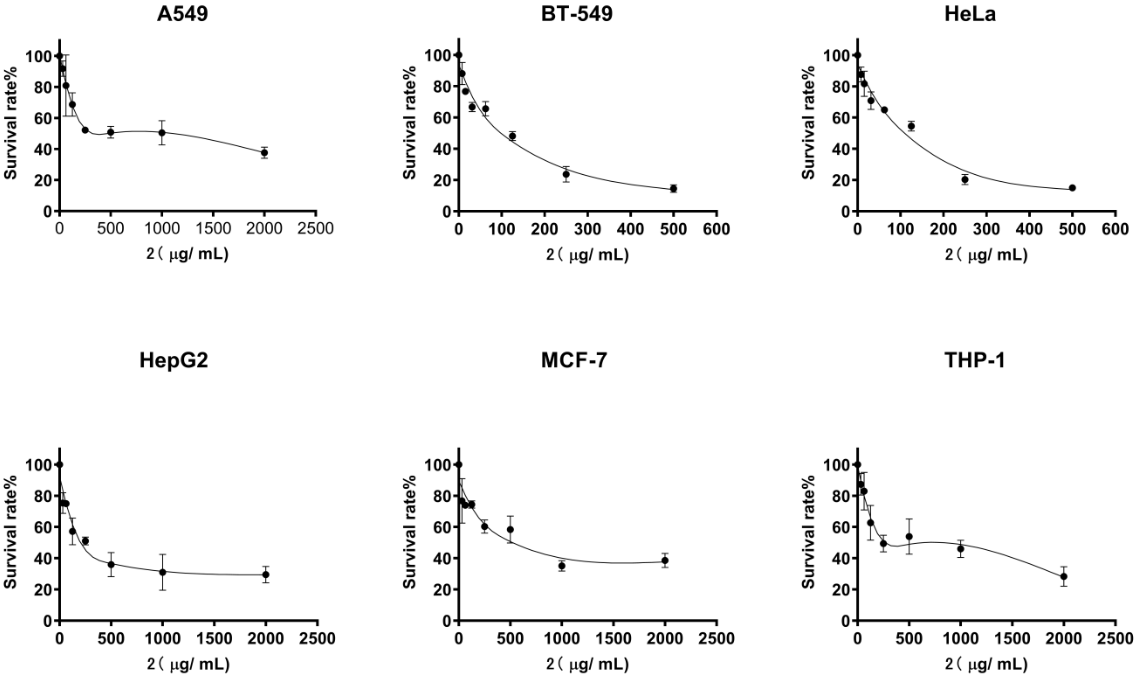

Figure S21. Cytotoxicity of compound **2**

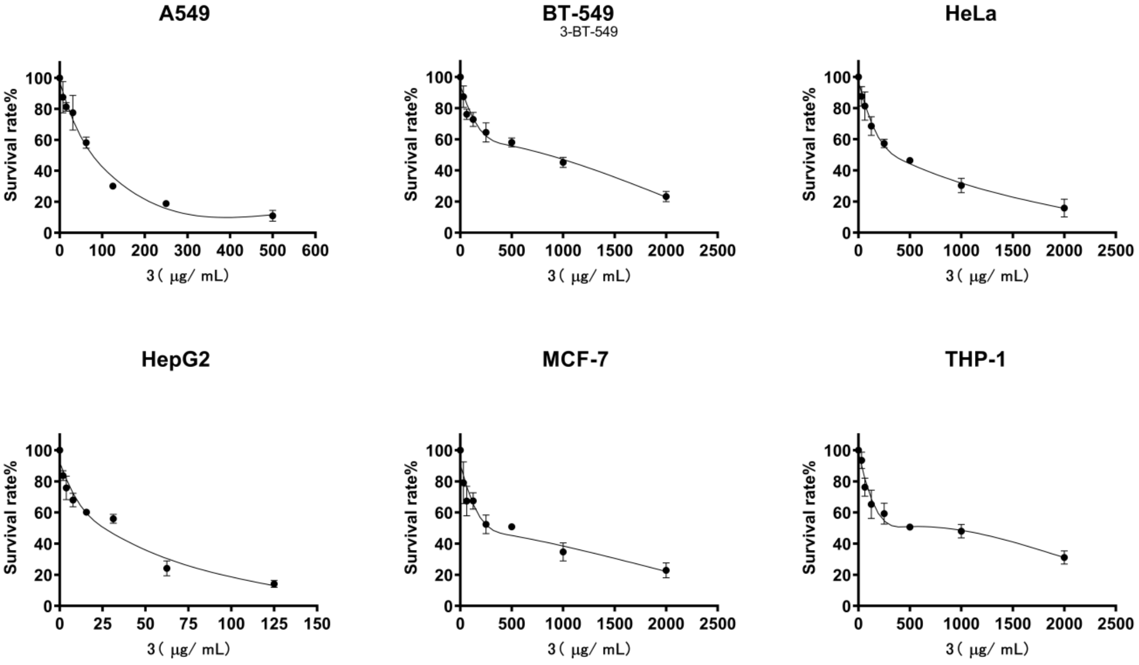

Figure S22. Cytotoxicity of compound **3**

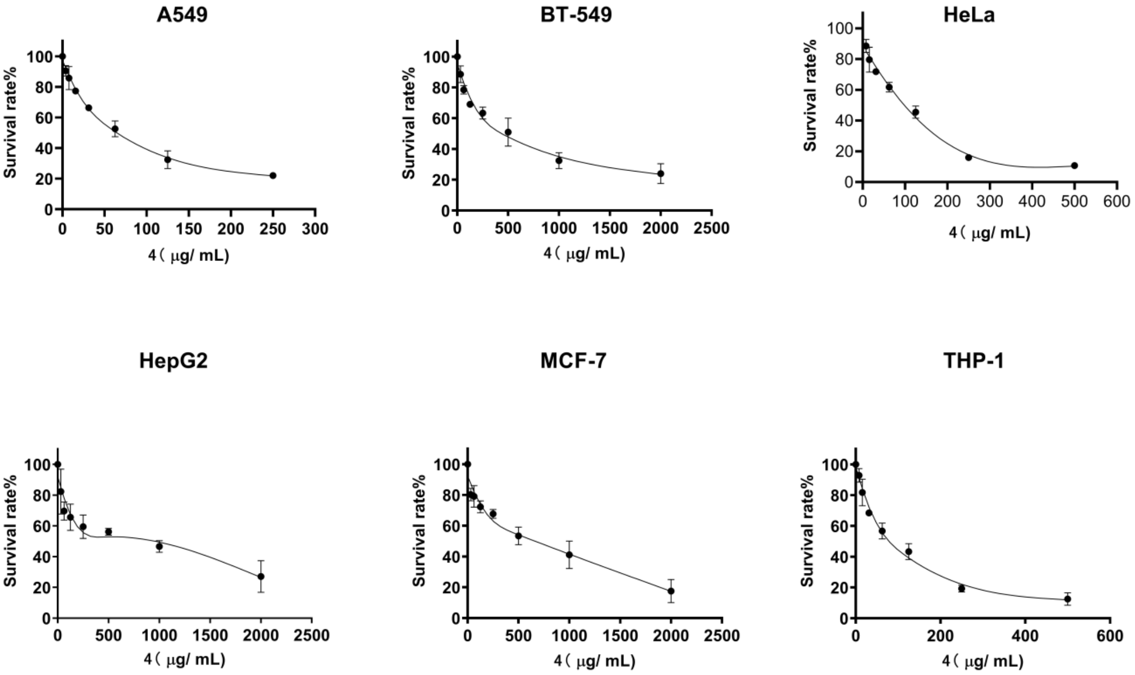

Figure S23. Cytotoxicity of compound **4**

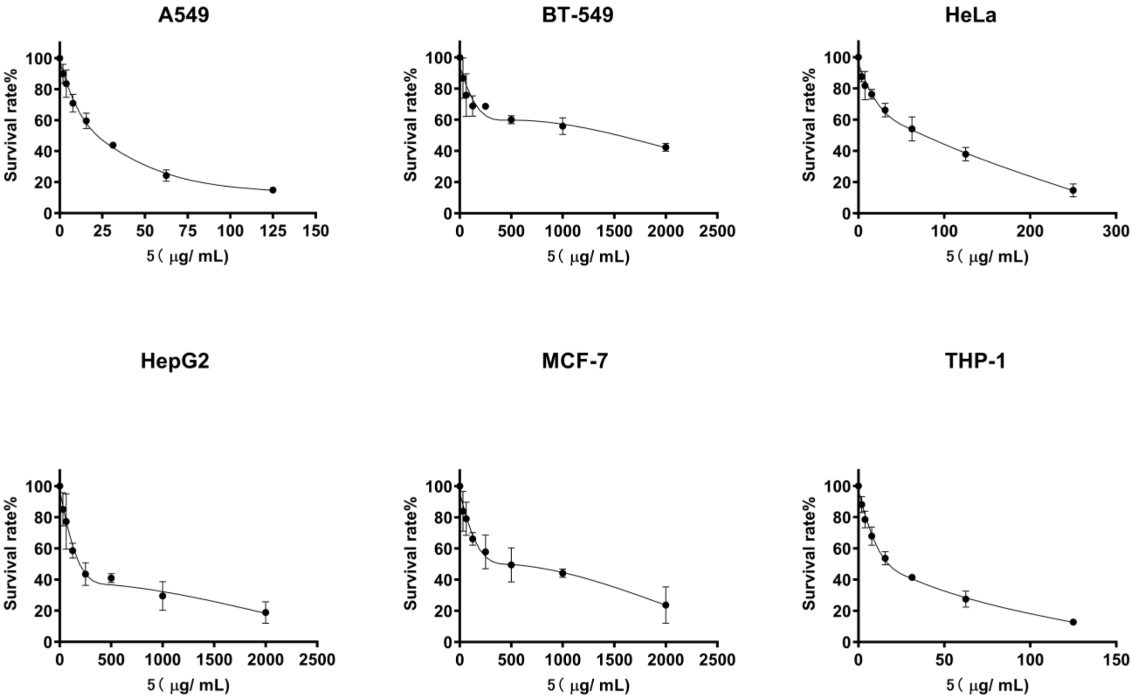

Figure S24. Cytotoxicity of compound **5**

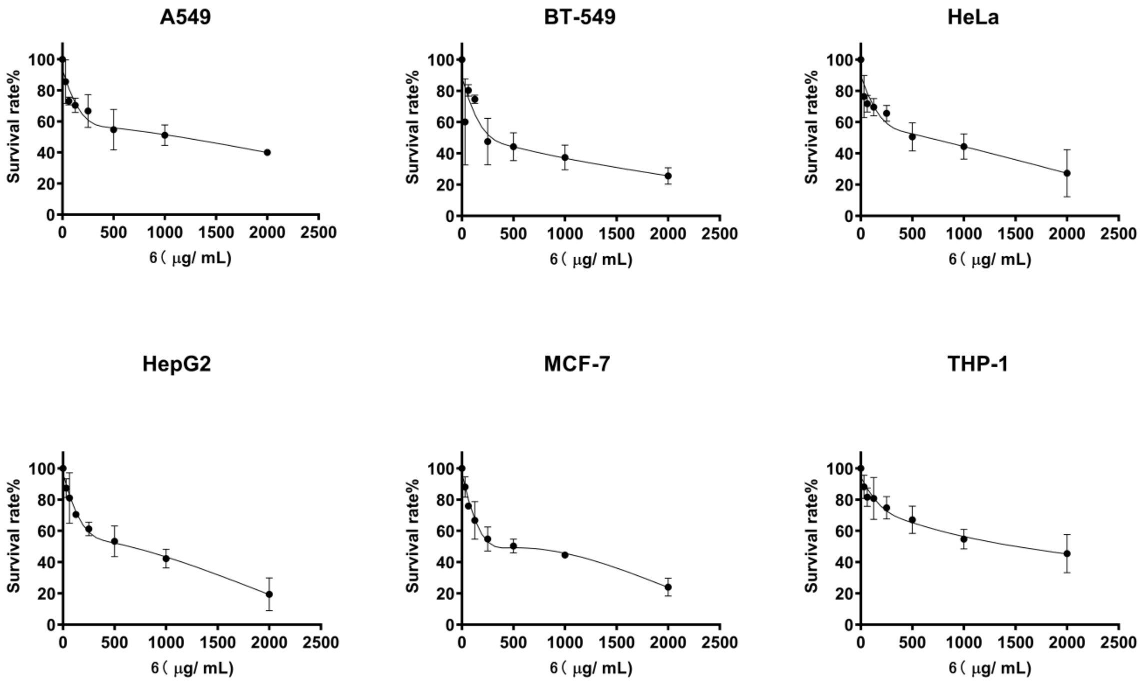

Figure S25. Cytotoxicity of compound **6**

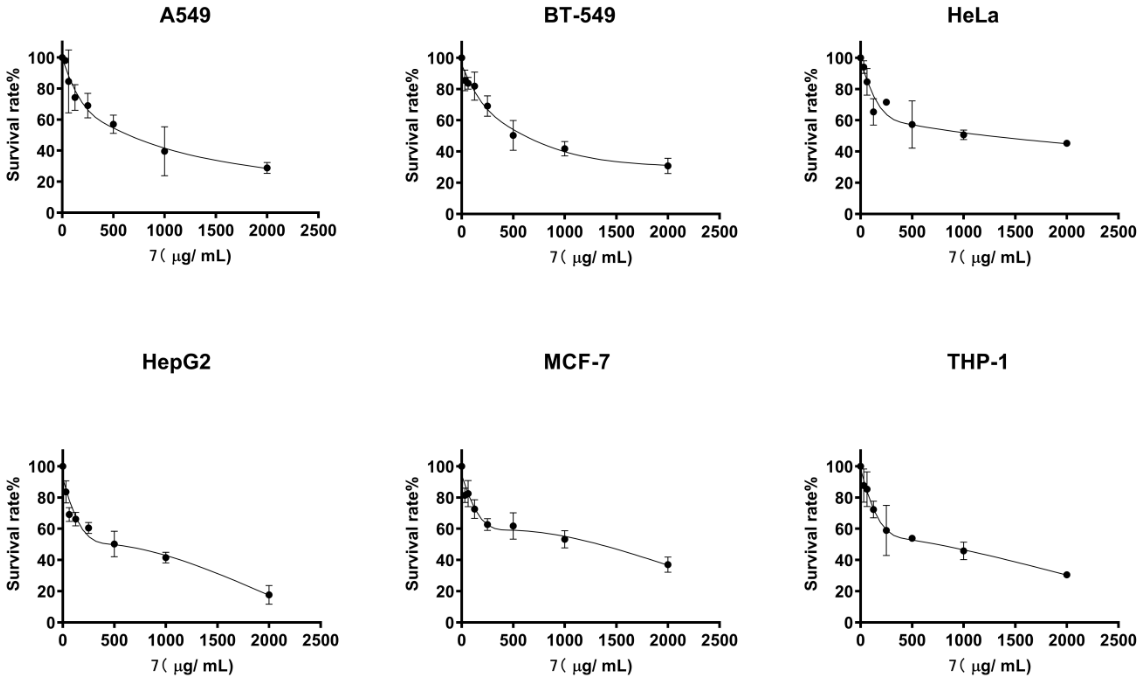

Figure S26. Cytotoxicity of compound **7**

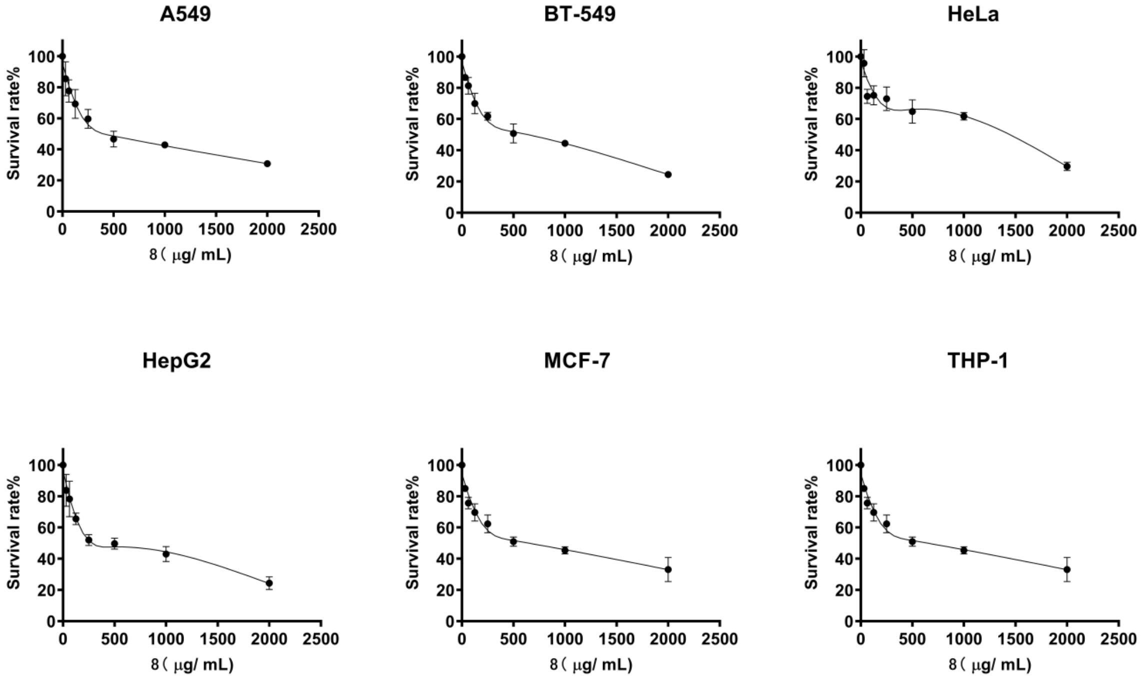

Figure S27. Cytotoxicity of compound **8**

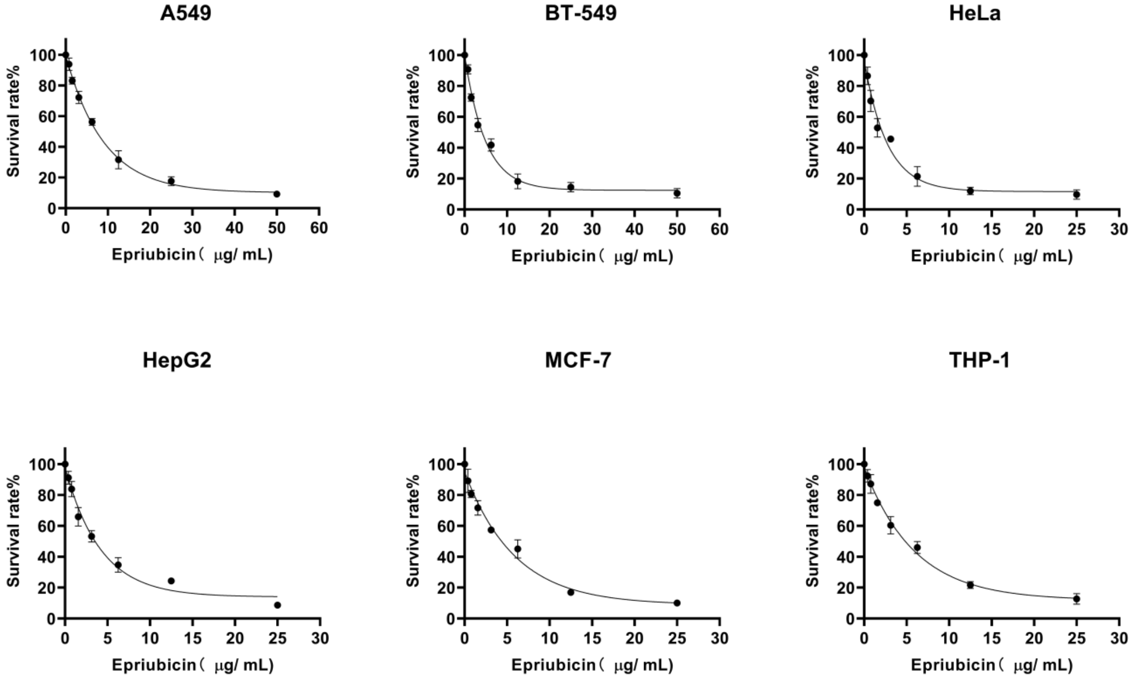

Figure S28. Cytotoxicity of epirubicin

Table S1. Conformational analysis of the B3LYP/6-31G(d) optimized conformers of **1** in the gas phase (T=298.15 K)

Conformer	E (Hartree)	C (Hartree)	G (kcal/mol)	ΔG (kcal/mol)	Population
**1**-1	-651.008652	0.158398	-408408.534275	0.0	36.29%
**1**-2	-651.008347	0.158549	-408408.248411	0.285863	22.39%
**1**-3	-651.008447	0.159247	-408407.872714	0.66156	11.87%
**1**-4	-651.009834	0.161116	-408407.570782	0.963493	7.13%
**1**-5	-651.007576	0.159194	-408407.359849	1.174425	4.99%
**1**-6	-651.006161	0.157847	-408407.317004	1.21727	4.64%
**1**-7	-651.00723	0.158951	-408407.29522	1.239055	4.48%
**1**-8	-651.008188	0.15995	-408407.269373	1.264902	4.29%
**1**-9	-651.006891	0.158738	-408407.216094	1.31818	3.92%

Electronic energy obtained at M062x/6-311+g(2d,p) level of theory; Thermal correction to Gibbs free energy obtained at B3LYP/6-31G(d) level of theory; Gibbs free energy (E + C); The relative Gibbs free energy; The Boltzmann distribution of each conformer.

Table S2. Atomic coordinates (Å) of **1**-1 obtained at the B3LYP/6-31G(d) level of theory in the gas phase.

C	2.208553	-0.029124	1.750979	H	-0.659149	-2.152758	-0.200933
C	2.476534	0.415416	0.317968	H	-3.352990	-2.018779	0.287016
C	1.588435	-0.307037	-0.717695	H	-4.356693	0.929007	0.323770
C	0.132842	-0.141010	-0.444171	H	-5.301926	-0.426379	-0.332491
C	-0.806637	-1.081650	-0.222075	H	-4.988043	-0.339155	1.398568
C	-2.080749	-0.417859	-0.020705	H	1.818974	0.151677	-1.694259
C	-3.293322	-0.934863	0.224490	H	2.314039	1.495164	0.213080
C	-4.546783	-0.142686	0.412440	H	2.859972	-1.730655	-0.720439
C	-0.528903	1.178002	-0.382326	H	4.421187	0.338770	0.599267
O	-0.076275	2.290406	-0.506420	H	2.940390	0.416057	2.436392
O	1.887181	-1.692347	-0.738915	H	1.214129	0.288447	2.079615
O	3.806304	0.066089	-0.097818	H	2.270165	-1.118739	1.827295
O	-1.877193	0.952378	-0.123137	-	-	-	-

Table S3. Atomic coordinates (Å) of **1**-2 obtained at the B3LYP/6-31G(d) level of theory in the gas phase.

C	2.189090	-0.206985	1.769617	H	-0.646481	-2.136730	-0.290490
C	2.468757	0.380616	0.396462	H	-3.339944	-2.035713	0.208592
C	1.592465	-0.238884	-0.723252	H	-4.979763	-0.409061	1.388722
C	0.133331	-0.111425	-0.456003	H	-4.360077	0.902733	0.360476
C	-0.799555	-1.066334	-0.269496	H	-5.299605	-0.432833	-0.343230
C	-2.076914	-0.417560	-0.040393	H	1.819320	0.324289	-1.648553
C	-3.286246	-0.949896	0.187353	H	2.310511	1.465356	0.404670
C	-4.543463	-0.172654	0.409213	H	2.882771	-1.641807	-0.754296
C	-0.535824	1.199393	-0.342533	H	4.104010	0.632067	-0.663742

O	-0.085114	2.316753	-0.428034	H	2.921327	0.168699	2.489174
O	1.916911	-1.606657	-0.884883	H	1.186926	0.069530	2.109396
O	3.836742	0.069143	0.080685	H	2.254472	-1.298600	1.736228
O	-1.881452	0.957566	-0.090613	-	-	-	-

Table S4. Atomic coordinates (Å) of **1**-3 obtained at the B3LYP/6-31G(d) level of theory in the gas phase.

C	-2.664957	-1.609240	-1.178322	H	0.059144	-1.845358	0.207524
C	-2.693359	-0.599396	-0.040395	H	2.732325	-2.339131	0.323460
C	-1.756197	0.592992	-0.242949	H	4.899433	-1.315034	-0.658254
C	-0.286146	0.276176	-0.183250	H	4.398611	0.269146	-0.023340
C	0.431943	-0.846358	0.031452	H	4.846984	-1.049617	1.082035
C	1.841489	-0.498888	0.021188	H	-1.975523	1.053013	-1.219565
C	2.921190	-1.279534	0.168607	H	-3.705183	-0.168919	0.024823
C	4.339066	-0.809033	0.138844	H	-1.491229	2.289422	0.676150
C	0.674269	1.393979	-0.270835	H	-2.304130	-0.581462	1.847758
O	0.457496	2.582108	-0.372607	H	-2.994635	-1.141313	-2.111991
O	-2.069075	1.516108	0.807606	H	-1.664403	-2.021112	-1.335615
O	-2.378619	-1.277076	1.171922	H	-3.342305	-2.436607	-0.947982
O	1.952338	0.874208	-0.173299	-	-	-	-

Table S5. Atomic coordinates (Å) of **1**-4 obtained at the B3LYP/6-31G(d) level of theory in the gas phase.

C	-2.276136	-0.371329	-1.899133	H	1.016945	2.296655	-0.574693
C	-2.660399	0.160338	-0.522750	H	3.668454	1.629643	-0.625225
C	-1.592988	1.083121	0.120837	H	5.065493	-0.514476	-1.024726
C	-0.174284	0.572216	0.036136	H	5.209435	-0.009628	0.656518
C	0.947528	1.259128	-0.270815	H	4.103284	-1.334207	0.226680
C	2.094745	0.389172	-0.116217	H	-1.618261	2.052616	-0.392185
C	3.401030	0.626571	-0.301643	H	-3.571508	0.763139	-0.604593
C	4.496494	-0.368502	-0.097232	H	-2.449108	0.498379	1.719322
C	0.256203	-0.798148	0.390979	H	-2.206135	-1.446414	0.492025
O	-0.389035	-1.783624	0.690275	H	-1.416528	-1.046667	-1.836899
O	-1.944397	1.302243	1.477986	H	-3.113146	-0.929247	-2.328564
O	-3.000308	-0.877885	0.392789	H	-2.017997	0.448660	-2.579800
O	1.630730	-0.856216	0.292845	-	-	-	-

Table S6. Atomic coordinates (Å) of **1**-5 obtained at the B3LYP/6-31G(d) level of theory in the gas phase.

C	2.409428	1.324830	1.129980	H	-0.468667	-2.127093	-0.200652
C	2.634356	0.009123	0.401878	H	-3.123200	-2.056986	0.429521
C	1.731714	-0.237646	-0.835616	H	-4.768143	-0.393127	1.546121

C	0.270645	-0.099259	-0.514214	H	-4.224904	0.855591	0.402073
C	-0.634412	-1.057731	-0.224532	H	-5.147869	-0.553719	-0.166227
C	-1.920674	-0.427886	0.006290	H	1.988521	0.497464	-1.607367
C	-3.103081	-0.974898	0.324297	H	3.666604	-0.053244	0.042867
C	-4.374027	-0.217751	0.536427	H	2.251884	-2.053155	-0.550154
C	-0.440683	1.198382	-0.495829	H	1.630591	-1.038948	1.709730
O	-0.039929	2.313684	-0.719171	H	2.467624	2.173191	0.442428
O	2.022440	-1.529915	-1.341338	H	1.417609	1.353992	1.595672
O	2.499361	-1.118931	1.279856	H	3.159703	1.445224	1.916793
O	-1.768041	0.941141	-0.160959	-	-	-	-

Table S7. Atomic coordinates (Å) of **1**-6 obtained at the B3LYP/6-31G(d) level of theory in the gas phase.

C	3.890114	-0.495889	-0.288485	H	-0.656365	-1.799336	1.264096
C	2.380673	-0.535555	-0.491329	H	-3.343855	-1.919133	0.787574
C	1.633136	0.093803	0.705089	H	-4.336253	0.589191	-0.768995
C	0.148408	0.068412	0.471460	H	-4.968969	-1.051853	-1.033418
C	-0.792727	-0.854516	0.753914	H	-5.286556	-0.229085	0.491318
C	-2.066215	-0.390497	0.236804	H	1.874140	-0.507375	1.589085
C	-3.280011	-0.957048	0.284964	H	2.111570	0.038529	-1.393876
C	-4.529415	-0.373226	-0.290659	H	1.663555	1.992905	0.311824
C	-0.519959	1.154769	-0.268852	H	2.372231	-2.309445	-1.309387
O	-0.053283	2.174298	-0.735253	H	4.165647	-1.081909	0.595618
O	2.095092	1.408769	0.965207	H	4.409433	-0.917885	-1.158163
O	1.891403	-1.873835	-0.589581	H	4.230127	0.532829	-0.146992
O	-1.860553	0.840898	-0.378637	-	-	-	-

Table S8. Atomic coordinates (Å) of **1**-7 obtained at the B3LYP/6-31G(d) level of theory in the gas phase.

C	2.153653	2.164561	0.465874	H	-0.653756	1.803390	-1.377095
C	2.532265	0.710319	0.240898	H	-3.302754	1.838037	-0.734028
C	1.746156	0.025760	-0.905017	H	-4.116153	-0.698621	0.879428
C	0.273693	-0.023507	-0.611320	H	-4.782591	0.922315	1.181903
C	-0.718401	0.859944	-0.849135	H	-5.167293	0.087210	-0.320557
C	-1.944492	0.352595	-0.263109	H	1.918351	0.600234	-1.823960
C	-3.176829	0.879008	-0.237166	H	3.590735	0.645593	-0.058144
C	-4.368751	0.256097	0.413721	H	1.749484	-1.907162	-0.598741
C	-0.324904	-1.145610	0.139548	H	2.537589	-0.910523	1.305807
O	0.186710	-2.178174	0.523805	H	2.286250	2.749467	-0.451417
O	2.281726	-1.277027	-1.121875	H	1.112117	2.245616	0.792194
O	2.319092	0.022425	1.465433	H	2.787048	2.595480	1.246377
O	-1.661698	-0.872979	0.331695	-	-	-	-

Table S9. Atomic coordinates (Å) of **1**-8 obtained at the B3LYP/6-31G(d) level of theory in the gas phase.

C	-2.171101	-0.028662	1.714183	H	0.821468	-2.150216	-0.880841
C	-2.385559	-0.866392	0.453064	H	3.487581	-1.995803	-0.331552
C	-1.595951	-0.349922	-0.780432	H	5.012327	-0.643010	1.260911
C	-0.125603	-0.210854	-0.520243	H	5.315588	-0.160025	-0.405356
C	0.878593	-1.111713	-0.577728	H	4.288736	0.855402	0.632160
C	2.108057	-0.465568	-0.160814	H	-1.761133	-1.074805	-1.584657
C	3.353696	-0.950974	-0.061665	H	-2.079168	-1.902323	0.645030
C	4.550570	-0.174625	0.381951	H	-1.753616	1.599090	-0.737053
C	0.464589	1.058794	-0.048581	H	-3.913723	-0.065722	-0.369137
O	-0.067696	2.131797	0.148119	H	-2.845961	-0.382927	2.498006
O	-2.179479	0.871235	-1.231678	H	-1.141554	-0.102270	2.082203
O	-3.754781	-0.911821	0.089301	H	-2.390824	1.027367	1.525485
O	1.814022	0.858132	0.152558	-	-	-	-

Table S10. Atomic coordinates (Å) of **1**-9 obtained at the B3LYP/6-31G(d) level of theory in the gas phase.

C	3.963201	-0.518425	-0.257028	H	-0.272795	-1.943623	0.047757
C	2.464152	-0.785296	-0.304534	H	-2.976603	-2.316633	-0.170806
C	1.653843	0.388162	0.272595	H	-4.509399	0.394562	-0.138400
C	0.162946	0.188253	0.153424	H	-4.991987	-0.999663	-1.130882
C	-0.608946	-0.916233	0.057932	H	-5.116461	-1.093644	0.623399
C	-1.997412	-0.503257	-0.016266	H	1.901932	0.462411	1.350653
C	-3.112715	-1.238752	-0.126732	H	2.144551	-0.934798	-1.340889
C	-4.503227	-0.695852	-0.195834	H	1.430190	2.266874	-0.174866
C	-0.747946	1.351045	0.156546	H	2.494925	-1.948613	1.262966
O	-0.492656	2.535523	0.235724	H	4.293784	-0.365216	0.779691
O	2.065900	1.562553	-0.407110	H	4.506316	-1.373560	-0.670587
O	2.124922	-2.002314	0.366639	H	4.213407	0.381351	-0.823190
O	-2.043321	0.885824	0.047814	-	-	-	-

Table S11. Key transitions, oscillator strengths, and rotatory strengths in the ECD spectrum of conformer **1**-1 at the CAM-B3LYP-SCRF/def2-SVP//B3LYP/6-31G(d) level of theory in MeOH with IEFPCM solvent model.

Num	transition	CI-coeff	ΔE (eV)	λ (nm)	f	Rvel	Rlen
1	49->50	0.70238	4.7278	262.25	0.7445	-3.2862	-5.7558
2	46->50	0.55281	5.0341	246.29	0.0035	12.7	14.8831
	47->50	0.30112					
	48->50	-0.23631					
3	45->50	0.27106	5.7221	216.68	0.0019	1.8153	1.8759
	48->50	0.58923					
4	45->50	0.62201	6.1831	200.52	0.0182	0.7449	-0.5741

	48->50	-0.25935					
5	46->50	-0.33973	6.5621	188.94	0.0008	-0.2531	-0.0141
	47->50	0.57252					
6	39->50	0.26906	7.1995	172.21	0.0023	4.6945	5.136
	42->50	-0.3713					
	44->50	0.476					
7	42->50	0.4785	7.3252	169.26	0.0027	-0.5791	-0.5321
	44->50	0.40432					
8	49->51	-0.24833	7.6444	162.19	0.2370	-10.7442	-9.4198
	49->52	0.60965					
9	39->50	0.28875	7.9534	155.89	0.0008	-3.7392	-3.0901
	49->53	0.44547					
10	39->50	-0.27411	7.9964	155.05	0.0150	-24.4275	-26.09
	41->50	-0.22944					
	49->53	0.36284					
11	41->50	-0.3098	8.0535	153.95	0.0311	57.3907	52.8407
	47->51	0.3191					
	49->53	-0.26571					
12	41->50	0.41899	8.0958	153.15	0.0883	-74.4355	-54.0173
	47->51	0.36858					
13	46->52	0.32812	8.1183	152.72	0.0015	-8.3068	-15.5841
	47->52	0.23084					
14	47->51	-0.30565	8.2518	150.25	0.0191	46.1239	44.8297
	48->51	-0.28151					
	49->51	0.35895					
15	43->50	0.49902	8.2817	149.71	0.0267	-9.1362	-8.0357
16	49->54	0.60681	8.4815	146.18	0.0701	-21.7666	-19.5525
17	37->50	0.5233	8.5418	145.15	0.0003	-3.1757	-2.8257
	43->50	0.31342					
18	48->51	0.30699	8.6659	143.07	0.0052	15.9749	16.466
	49->51	0.32406					
	49->55	0.3517					
19	49->55	0.52563	8.8151	140.65	0.0024	4.2452	6.0344
20	39->50	0.33844	8.8619	139.91	0.0004	2.6934	2.0507
	40->50	0.58652					
21	33->50	0.32573	8.9683	138.25	0.0016	1.2367	1.1995
	35->50	0.44831					
22	45->52	0.22427	9.1157	136.01	0.0581	-27.0562	-23.9009
	48->52	0.49732					
23	35->50	-0.28234	9.2415	134.16	0.0012	4.7053	5.5494
	38->50	0.54857					
24	49->56	0.55567	9.3409	132.73	0.0116	-21.7405	-22.9771
25	49->57	0.36547	9.4670	130.96	0.0229	22.7911	28.5063

	49->58	0.22702					
	49->60	-0.30871					
26	33->50	0.41504	9.5362	130.01	0.0165	-32.9852	-33.4439
	35->50	-0.22635					
27	33->50	-0.27154	9.5550	129.76	0.0201	54.8909	61.8075
	45->52	0.27523					
28	47->52	0.24174	9.6057	129.07	0.0045	-0.3372	-5.0815
	49->58	0.38876					
29	45->52	0.35918	9.6616	128.33	0.0623	56.3426	57.5904
	48->54	-0.236					
30	36->50	0.6444	9.7092	127.70	0.0043	-9.167	-6.3581

Number of the excited states; Only transitions with contribution over 10.0% were listed; Configuration-interaction coefficient; Excitation energy; Wavelength; Oscillator strength; Rotatory strength in length form (10-40 cgs); Rotatory strength in velocity form (10-40 cgs).

Table S12. Key transitions, oscillator strengths, and rotatory strengths in the ECD spectrum of conformer **1**-2 at the CAM-B3LYP-SCRF/def2-SVP//B3LYP/6-31G(d) level of theory in MeOH with IEFPCM solvent model.

Num	transition	CI-coeff	ΔE (eV)	λ (nm)	f	Rvel	Rlen
1	49->50	0.70157	4.7027	263.65	0.7416	-4.2135	-7.0126
2	47->50	0.61931	5.0351	246.24	0.0071	18.4287	20.8743
	48->50	-0.22889					
3	45->50	-0.23403	5.6460	219.60	0.0025	2.5051	2.625
	47->50	0.24652					
	48->50	0.60977					
4	45->50	0.48244	6.1969	200.08	0.0178	-2.2395	-3.9625
	46->50	0.45831					
5	45->50	-0.41007	6.6534	186.35	0.0062	2.3396	3.3415
	46->50	0.47926					
6	39->50	-0.26359	7.2867	170.15	0.0004	-1.785	-1.9715
	42->50	0.59504					
7	49->51	0.47155	7.5827	163.51	0.1987	-24.9056	-25.8823
	49->52	-0.42969					
8	43->50	0.47238	7.7080	160.85	0.0146	0.3522	0.6265
	44->50	-0.35303					
9	48->51	-0.3734	7.7909	159.14	0.0458	15.2731	20.0547
	49->51	0.24017					
	49->52	0.41647					
10	39->50	-0.23771	7.9396	156.16	0.0044	-17.9401	-17.9092
	42->50	-0.22651					
	43->50	0.3447					
	44->50	0.37403					
	49->53	-0.27158					

11	44->50	0.35052	7.9616	155.73	0.0060	8.0832	8.0712
	49->53	0.42582					
12	39->50	-0.28743	8.0170	154.65	0.0034	7.8295	4.311
	40->50	-0.24222					
	42->50	-0.23468					
	49->53	0.39453					
13	41->50	0.54908	8.0889	153.28	0.1160	49.186	52.0001
14	34->50	-0.22998	8.1813	151.55	0.0261	-13.9409	-10.3996
	41->50	-0.23439					
	47->51	-0.23047					
	47->52	0.32557					
15	41->50	-0.22842	8.4302	147.07	0.0664	3.7971	0.8069
	49->54	0.42492					
	49->55	-0.31125					
16	36->50	0.43261	8.4761	146.27	0.0030	-6.9503	-6.4423
	37->50	-0.26098					
	40->50	0.30965					
17	46->51	0.43291	8.5433	145.12	0.0108	-32.9315	-25.9463
	49->54	-0.25101					
18	48->51	0.26703	8.6276	143.71	0.0093	22.5195	23.5516
	49->51	0.25586					
	49->55	-0.25597					
	49->56	0.34618					
19	49->54	-0.29105	8.8057	140.80	0.0037	12.0098	12.4658
	49->55	-0.30288					
	49->56	0.34375					
	49->57	0.25754					
20	34->50	0.39702	8.9172	139.04	0.0029	2.5798	2.8016
	36->50	0.2333					
	39->50	0.23484					
	40->50	-0.30083					
21	34->50	-0.26318	8.9829	138.02	0.0394	-50.9657	-46.903
	48->52	0.38515					
22	34->50	-0.2802	9.0346	137.23	0.0221	26.53	27.7124
	38->50	0.33359					
23	38->50	0.58909	9.1153	136.02	0.0090	-9.0813	-8.4644
	39->50	-0.2472					
24	49->55	0.36077	9.1908	134.90	0.0116	4.931	8.8159
	49->56	0.29304					
25	48->52	0.24475	9.2822	133.57	0.0322	-39.475	-36.2506
	49->56	-0.27308					
	49->57	0.44264					
26	33->50	-0.34016	9.4897	130.65	0.0023	-7.5924	-7.9767

	37->50	0.51427					
27	45->52	0.26271	9.5290	130.11	0.0301	64.252	61.6914
	47->51	-0.23192					
28	44->51	-0.25479	9.6387	128.63	0.0606	10.8979	10.612
	47->51	0.30226					
29	46->51	0.26494	9.6665	128.26	0.0099	5.1128	-3.4021
	49->59	-0.2243					
30	45->51	0.25955	9.6859	128.00	0.0024	-9.848	-0.0651
	48->57	0.26637					

Number of the excited states; Only transitions with contribution over 10.0% were listed; Configuration-interaction coefficient; Excitation energy; Wavelength; Oscillator strength; Rotatory strength in length form (10-40 cgs); Rotatory strength in velocity form (10-40 cgs).

Table S13. Key transitions, oscillator strengths, and rotatory strengths in the ECD spectrum of conformer **1**-3 at the CAM-B3LYP-SCRF/def2-SVP//B3LYP/6-31G(d) level of theory in MeOH with IEFPCM solvent model.

Num	transition	CI-coeff	ΔE (eV)	λ (nm)	f	Rvel	Rlen
1	49->50	0.70059	4.7555	260.72	0.6963	-19.4859	-20.4995
2	45->50	-0.22789	5.0407	245.96	0.0051	14.0424	13.3512
	46->50	0.49501					
	47->50	0.24877					
	48->50	-0.31442					
3	47->50	0.46967	5.7536	215.49	0.0057	2.0744	2.3549
	48->50	0.48888					
4	46->50	-0.39442	5.8867	210.62	0.0034	2.2276	3.1424
	47->50	0.44571					
	48->50	-0.34478					
5	45->50	0.64048	5.9455	208.53	0.0132	4.2915	5.4565
6	38->50	-0.23712	7.1946	172.33	0.0022	4.3596	4.1139
	41->50	0.32866					
	42->50	0.33736					
	44->50	0.41291					
7	41->50	-0.28941	7.5076	165.14	0.0086	-12.8236	-12.4918
	42->50	-0.27984					
	44->50	0.53544					
8	49->51	0.65206	7.5635	163.92	0.2018	20.7174	21.1178
9	39->50	-0.27121	7.8703	157.53	0.0082	5.8864	5.6432
	40->50	0.2365					
	43->50	0.49038					
10	41->50	-0.28971	7.9138	156.67	0.0281	37.04	40.9075
	46->51	0.33116					
11	40->50	0.23397	7.9892	155.19	0.0457	-20.6682	-16.9643
	42->50	0.2828					

	43->50	-0.25171					
12	49->53	0.56358	8.0765	153.51	0.0271	-14.8364	-21.5767
13	36->50	-0.24231	8.1890	151.40	0.0127	-25.7227	-29.6668
	38->50	0.30124					
14	48->51	0.37155	8.2384	150.50	0.0281	-16.2832	-40.5296
	48->52	-0.29916					
15	49->52	-0.31397	8.3166	149.08	0.1633	56.9532	57.5789
	49->54	0.38857					
16	46->51	-0.22772	8.4075	147.47	0.0113	5.3648	9.397
	47->51	0.52401					
17	40->50	0.4253	8.5328	145.30	0.0029	5.997	8.1146
	41->50	0.23627					
	42->50	-0.24615					
18	36->50	0.54722	8.6856	142.75	0.0008	3.5499	4.9448
	43->50	-0.25518					
19	49->52	0.37022	8.8192	140.58	0.0198	-17.7535	-19.0087
	49->54	0.34269					
	49->55	-0.30855					
20	49->55	-0.32255	8.8712	139.76	0.0021	5.4852	5.1788
	49->56	0.50068					
21	34->50	0.45146	9.0331	137.25	0.0055	4.1268	3.7462
22	39->50	-0.22453	9.1294	135.81	0.0271	0.7486	1.8378
	46->51	0.2263					
	48->51	0.27634					
	48->52	0.34115					
	48->54	0.28415					
23	38->50	0.27578	9.1884	134.94	0.0286	-9.618	-9.7849
	39->50	0.37155					
	45->51	0.35898					
24	38->50	-0.26312	9.2221	134.44	0.0149	-5.4873	-6.5902
	39->50	-0.2798					
	45->51	0.41527					
25	47->52	0.33844	9.3199	133.03	0.0008	2.7382	0.7007
	47->55	-0.25046					
	49->55	-0.29242					
	49->56	-0.25243					
26	37->50	0.62293	9.3708	132.31	0.0005	2.479	3.9171
27	48->56	0.23185	9.3920	132.01	0.0096	-15.786	-16.964
28	47->52	0.27868	9.4299	131.48	0.0465	0.3412	-0.6954
	48->55	-0.23181					
	49->55	0.25121					
29	49->57	-0.32342	9.5902	129.28	0.0034	-11.9012	-7.753
	49->58	0.39541					

30	35->50	0.52283	9.6346	128.69	0.0218	-2.7698	-2.612

Number of the excited states; Only transitions with contribution over 10.0% were listed; Configuration-interaction coefficient; Excitation energy; Wavelength; Oscillator strength; Rotatory strength in length form (10-40 cgs); Rotatory strength in velocity form (10-40 cgs).

Table S14. Key transitions, oscillator strengths, and rotatory strengths in the ECD spectrum of conformer **1**-4 at the CAM-B3LYP-SCRF/def2-SVP//B3LYP/6-31G(d) level of theory in MeOH with IEFPCM solvent model.

Num	transition	CI-coeff	ΔE (eV)	λ (nm)	f	Rvel	Rlen
1	49->50	0.7019	4.7181	262.78	0.7632	-2.3063	0.1248
2	46->50	0.43147	5.1042	242.91	0.0055	-14.0339	-15.0671
	47->50	0.4601					
3	47->50	0.24982	5.4879	225.92	0.0032	1.893	1.2013
	48->50	0.6434					
4	45->50	0.49693	5.8452	212.11	0.0135	-4.3088	-4.0984
	46->50	0.2865					
	47->50	-0.35884					
5	44->50	0.25415	6.3652	194.78	0.0066	2.8802	3.1404
	45->50	0.43689					
	46->50	-0.34488					
	47->50	0.27821					
6	44->50	0.54344	6.9687	177.92	0.0020	-3.22	-4.1269
	46->50	0.25071					
7	39->50	0.27192	7.2716	170.51	0.0025	5.3764	5.6744
	41->50	0.45631					
	42->50	0.28295					
	44->50	0.25524					
8	43->50	0.63745	7.5474	164.27	0.0142	-4.9778	-4.9367
9	49->51	0.65031	7.6180	162.75	0.2049	1.0848	-2.6216
10	39->50	0.52327	7.8735	157.47	0.0055	10.6914	11.3806
	41->50	-0.34395					
11	40->50	0.48595	8.0223	154.55	0.1107	-10.4693	-8.0744
	42->50	-0.31944					
12	49->52	0.42267	8.0839	153.37	0.0033	1.0003	-2.7795
	49->53	0.48281					
13	46->51	0.34334	8.1715	151.73	0.0128	7.0654	5.0987
	47->51	0.33754					
14	37->50	0.26493	8.4068	147.48	0.0249	30.9587	34.4852
	42->50	0.42227					
	49->54	-0.28857					
15	37->50	0.24868	8.4520	146.69	0.0792	-49.2831	-55.9939
	49->54	0.50361					
16	48->51	0.45422	8.5198	145.53	0.0173	-2.5335	-3.3901

	48->54	0.24747					
17	37->50	-0.29119	8.5455	145.09	0.0217	20.8864	21.4867
	40->50	0.30007					
	42->50	0.26936					
18	49->52	0.44255	8.6835	142.78	0.0087	-17.0244	-16.421
	49->53	-0.4008					
19	48->51	0.2786	8.8233	140.52	0.0093	14.8208	14.0229
	48->52	0.54403					
20	49->55	0.59923	8.9053	139.23	0.0020	-0.4631	0.7701
21	34->50	0.24077	8.9278	138.87	0.0055	-10.8939	-13.4204
	35->50	0.32849					
	37->50	0.3267					
22	45->51	0.32933	9.1300	135.80	0.0593	27.5868	27.2419
	46->51	0.313					
	47->51	-0.32248					
23	47->52	0.43423	9.2526	134.00	0.0159	32.8363	39.5971
24	38->50	0.67292	9.3706	132.31	0.0022	-0.7257	-0.7219
25	48->57	0.28474	9.3943	131.98	0.0062	26.0358	16.2753
	49->56	0.35291					
	49->57	-0.3136					
26	48->57	-0.32345	9.4867	130.69	0.0062	-0.9886	-1.4934
	49->56	0.39595					
27	45->51	0.49298	9.5215	130.22	0.0298	16.4424	23.3313
28	34->50	0.24644	9.5385	129.98	0.0016	1.5301	0.696
	36->50	0.46473					
	37->50	-0.22623					
29	33->50	0.30088	9.6609	128.34	0.0060	15.593	16.9401
	34->50	0.39643					
	35->50	-0.23517					
	36->50	-0.27123					
30	46->52	0.29981	9.7279	127.45	0.0194	-26.2099	-27.678
	49->57	0.22896					
	49->58	0.25328					
	49->59	-0.25905					

Number of the excited states; Only transitions with contribution over 10.0% were listed; Configuration-interaction coefficient; Excitation energy; Wavelength; Oscillator strength; Rotatory strength in length form (10-40 cgs); Rotatory strength in velocity form (10-40 cgs).

Table S15. Key transitions, oscillator strengths, and rotatory strengths in the ECD spectrum of conformer **1**-5 at the CAM-B3LYP-SCRF/def2-SVP//B3LYP/6-31G(d) level of theory in MeOH with IEFPCM solvent model.

Num	transition	CI-coeff	ΔE (eV)	λ (nm)	f	Rvel	Rlen
1	49->50	0.69974	4.7093	263.27	0.7188	-11.8799	-16.0635

2	46->50	0.41858	4.9664	249.64	0.0123	18.9343	21.2354
	47->50	0.33888					
	48->50	0.40806					
3	46->50	-0.4788	5.7578	215.33	0.0057	8.2475	10.1724
	48->50	0.49665					
4	45->50	-0.25899	5.8517	211.88	0.0101	0.8569	-0.1732
	46->50	-0.23086					
	47->50	0.54554					
	48->50	-0.24692					
5	45->50	0.60766	6.7212	184.47	0.0123	-2.5541	-1.8914
	47->50	0.26561					
6	39->50	-0.31275	7.1859	172.54	0.0003	1.0481	0.7987
	41->50	0.4481					
	42->50	0.36083					
7	44->50	0.62937	7.4397	166.65	0.0254	-5.1886	-5.7171
8	49->51	0.59413	7.6183	162.74	0.1881	-1.3927	-0.0158
9	41->50	-0.27246	7.7287	160.42	0.0072	4.9241	6.1117
	43->50	0.56588					
10	39->50	0.27424	7.9251	156.45	0.0031	0.1288	-0.3552
	49->52	0.35807					
	49->54	0.29602					
11	39->50	0.26087	7.9476	156.00	0.0356	-44.1521	-49.9941
	40->50	0.29666					
	42->50	0.30532					
	49->52	-0.24477					
12	41->50	0.34243	8.0516	153.99	0.1203	10.2884	23.2596
	42->50	-0.31921					
	49->52	-0.25702					
	49->54	-0.22674					
13	39->50	0.29831	8.1500	152.13	0.0053	-23.2889	-26.9817
	46->51	0.22418					
	48->51	0.26753					
14	47->51	-0.27958	8.2705	149.91	0.0048	0.2703	-1.2287
	48->51	0.25498					
	49->52	-0.2292					
	49->54	0.33798					
15	49->53	0.51938	8.3582	148.34	0.0720	-37.0937	-35.7142
16	48->51	-0.27831	8.4421	146.86	0.0502	77.7626	73.8897
	49->52	-0.28932					
	49->54	0.31959					
17	37->50	0.43684	8.5054	145.77	0.0023	4.0573	3.4822
	40->50	0.39751					
18	37->50	-0.36993	8.6827	142.79	0.0058	7.2555	10.1811

	39->50	-0.25719					
	40->50	0.37555					
19	49->55	0.53991	8.7196	142.19	0.0058	1.6946	0.818
20	34->50	0.30068	8.8271	140.46	0.0013	-5.8197	-5.5346
	35->50	0.40413					
21	45->51	0.22528	8.8572	139.98	0.0099	-13.68	-6.0136
	47->51	0.27325					
22	46->51	-0.23074	9.1201	135.95	0.0052	-3.6217	-2.8632
	48->51	0.3017					
	48->53	0.34993					
23	47->51	0.34194	9.2075	134.66	0.0769	-59.9042	-57.0748
	49->57	-0.27969					
24	49->55	-0.23779	9.2557	133.96	0.0080	4.3375	-2.4386
	49->56	0.37064					
	49->57	0.35765					
25	38->50	0.30098	9.3866	132.09	0.0223	6.3494	5.2042
	49->56	0.30424					
26	38->50	0.45854	9.4235	131.57	0.0194	35.8571	39.2795
	49->56	-0.25798					
27	34->50	-0.27525	9.6130	128.98	0.0037	-2.5905	-4.1758
	36->50	0.48875					
	38->50	-0.26319					
28	45->51	-0.22676	9.6473	128.52	0.0327	49.1088	48.8639
	48->53	0.26296					
29	48->52	0.24826	9.7032	127.78	0.0074	-3.6929	-15.1556
	48->53	0.22834					
	49->59	0.25341					
30	47->52	0.28588	9.7415	127.27	0.0167	0.8748	0.3729
	48->55	0.31836					

Number of the excited states; Only transitions with contribution over 10.0% were listed; Configuration-interaction coefficient; Excitation energy; Wavelength; Oscillator strength; Rotatory strength in length form (10-40 cgs); Rotatory strength in velocity form (10-40 cgs).

Table S16. Key transitions, oscillator strengths, and rotatory strengths in the ECD spectrum of conformer **1**-6 at the CAM-B3LYP-SCRF/def2-SVP//B3LYP/6-31G(d) level of theory in MeOH with IEFPCM solvent model.

Num	transition	CI-coeff	ΔE (eV)	λ (nm)	f	Rvel	Rlen
1	49->50	0.69991	4.7111	263.17	0.7232	7.4781	12.0154
2	46->50	0.62411	5.0611	244.97	0.0145	-20.9401	-21.6346
3	47->50	0.4476	5.5899	221.80	0.0098	-3.8332	-3.9883
	48->50	0.51813					
4	45->50	-0.41935	5.8050	213.58	0.0110	0.6793	0.8464
	47->50	0.46886					

	48->50	-0.30141					
5	45->50	0.50371	6.0475	205.02	0.0132	-2.1748	-2.5885
	46->50	-0.25475					
	47->50	0.23002					
	48->50	-0.33958					
6	38->50	0.24512	7.2112	171.93	0.0023	6.9536	7.2548
	42->50	-0.35759					
	43->50	0.48029					
7	42->50	0.46287	7.3410	168.89	0.0061	-3.3653	-3.5032
	43->50	0.38699					
8	49->51	0.63404	7.6370	162.35	0.2562	-15.5787	-18.1613
9	44->50	0.64898	7.7397	160.19	0.0127	-0.3949	-0.4127
10	38->50	0.46854	7.9464	156.02	0.0019	0.2833	-1.3507
	42->50	0.26548					
	46->51	0.23192					
11	47->52	-0.27755	7.9795	155.38	0.0163	21.6254	15.9594
	48->51	0.25711					
	48->52	0.37896					
12	49->53	0.55106	8.0569	153.88	0.0210	-15.6741	-25.2327
13	41->50	0.40915	8.0699	153.64	0.0990	-26.5866	-22.613
	49->53	0.26772					
14	38->50	-0.26196	8.1976	151.24	0.0039	10.0777	12.1665
	46->51	0.37789					
15	41->50	0.23247	8.4101	147.42	0.1057	-29.6639	-32.5029
	49->54	0.58116					
16	36->50	0.24631	8.4918	146.00	0.0025	7.48	3.8579
	37->50	0.32727					
	40->50	0.36168					
17	47->51	0.38541	8.5683	144.70	0.0063	0.2578	8.8274
	48->51	0.30741					
18	49->52	0.25978	8.7753	141.29	0.0048	-6.6937	-4.926
	49->56	0.3919					
19	35->50	-0.22449	8.8199	140.57	0.0028	0.6792	1.8283
	36->50	0.25529					
	39->50	0.25986					
	49->52	0.22822					
	49->56	0.24053					
20	49->52	0.38269	8.8565	139.99	0.0069	-10.0643	-8.0761
	49->55	-0.29143					
	49->56	-0.2986					
21	37->50	-0.36919	9.0197	137.46	0.0117	10.8349	10.8808
	40->50	0.41698					
22	39->50	-0.2828	9.0665	136.75	0.0082	12.1957	11.9896

	49->55	0.40172					
	49->56	-0.22531					
23	34->50	0.24864	9.1078	136.13	0.0022	2.0094	2.1387
	39->50	0.43233					
	49->55	0.22652					
24	47->52	0.25935	9.1641	135.29	0.0496	-25.1749	-37.9515
	48->51	0.3646					
25	47->51	0.31481	9.2201	134.47	0.0152	29.9829	32.3656
	48->52	0.33553					
26	49->57	0.50445	9.3080	133.20	0.0035	-5.4013	-13.7287
27	45->51	0.47096	9.3727	132.28	0.0456	11.3965	17.7735
	47->52	-0.26032					
28	47->55	0.43591	9.5032	130.47	0.0086	20.2272	14.7378
29	48->55	-0.31141	9.6157	128.94	0.0182	28.1813	22.9312
	49->58	0.33395					
30	35->50	0.42863	9.6397	128.62	0.0192	3.3093	4.4522
	36->50	0.43173					

Number of the excited states; Only transitions with contribution over 10.0% were listed; Configuration-interaction coefficient; Excitation energy; Wavelength; Oscillator strength; Rotatory strength in length form (10-40 cgs); Rotatory strength in velocity form (10-40 cgs).

Table S17. Key transitions, oscillator strengths, and rotatory strengths in the ECD spectrum of conformer **1**-7 at the CAM-B3LYP-SCRF/def2-SVP//B3LYP/6-31G(d) level of theory in MeOH with IEFPCM solvent model.

Num	transition	CI-coeff	ΔE (eV)	λ (nm)	f	Rvel	Rlen
1	49->50	0.69692	4.6619	265.95	0.6458	30.4764	37.1697
2	46->50	0.3543	5.0834	243.90	0.0350	-32.8103	-35.3847
	47->50	0.50932					
3	48->50	0.66584	5.2954	234.14	0.0396	-11.3391	-13.1991
4	45->50	0.6644	5.9527	208.28	0.0166	-7.2909	-7.8614
5	46->50	0.5658	6.3323	195.80	0.0006	4.6748	4.5713
	47->50	-0.40785					
6	43->50	0.42877	7.0703	175.36	0.0020	4.9923	5.0835
	44->50	-0.41496					
7	41->50	0.53198	7.3710	168.21	0.0043	-4.4349	-4.6582
	44->50	-0.28537					
8	49->51	0.6362	7.6060	163.01	0.2281	5.0409	1.6024
9	43->50	0.47442	7.8051	158.85	0.0290	1.3182	0.2791
	44->50	0.43911					
10	40->50	-0.26454	7.9153	156.64	0.0438	7.5882	6.7518
	41->50	0.2248					
	42->50	0.24646					
	49->52	0.32087					

11	38->50	0.34581	7.9322	156.30	0.0022	12.5049	11.1752
	49->52	0.30147					
12	38->50	0.23423	8.0479	154.06	0.0611	-36.783	-49.2276
	40->50	-0.22546					
	42->50	0.34606					
	49->52	-0.22642					
	49->53	-0.31677					
13	48->52	-0.32713	8.0887	153.28	0.0208	14.6047	18.9611
	48->54	0.23855					
	49->53	0.34847					
	49->54	0.23218					
14	34->50	-0.2844	8.1689	151.78	0.0156	32.6414	38.1388
	37->50	0.23582					
	47->51	0.29879					
15	42->50	-0.23978	8.3226	148.97	0.0995	-55.2516	-60.7979
	49->52	0.22739					
	49->53	-0.3049					
	49->54	0.37316					
16	40->50	0.34491	8.4239	147.18	0.0183	-4.8886	-2.542
	42->50	0.24671					
	48->51	0.42158					
17	40->50	-0.33481	8.4928	145.99	0.0255	26.4797	33.6594
	48->51	0.3657					
18	36->50	0.48767	8.6421	143.46	0.0011	12.5094	12.9494
	37->50	0.34512					
	42->50	0.22543					
19	49->52	0.23409	8.7410	141.84	0.0163	-14.6288	-14.454
	49->55	0.49452					
20	34->50	0.29343	8.8498	140.10	0.0018	3.4303	2.6508
	37->50	-0.24503					
	38->50	0.27173					
21	49->54	0.30256	8.8721	139.75	0.0020	6.2414	4.0974
	49->55	0.24917					
	49->56	-0.27043					
22	34->50	0.25913	8.9523	138.49	0.0024	1.819	1.0283
	39->50	0.59018					
23	46->52	-0.25695	9.0315	137.28	0.0217	-15.9481	-13.8884
	46->54	0.22822					
	48->52	0.25814					
24	49->56	0.44748	9.1058	136.16	0.0060	11.5876	5.0059
25	34->50	0.37415	9.1490	135.52	0.0018	-0.013	-0.0414
	37->50	0.3652					
26	45->51	0.41796	9.3271	132.93	0.0208	-24.8417	-23.6762

	48->54	-0.22399					
27	46->51	0.45172	9.4244	131.56	0.0047	-10.1538	-8.1611
	47->51	-0.35262					
28	45->51	0.39935	9.4560	131.12	0.0766	7.5611	8.9747
29	48->55	0.25178	9.5041	130.45	0.0114	18.855	14.0807
	48->56	0.23559					
	49->57	0.4197					
30	35->50	0.60057	9.5972	129.19	0.0321	3.8452	4.7608

Number of the excited states; Only transitions with contribution over 10.0% were listed; Configuration-interaction coefficient; Excitation energy; Wavelength; Oscillator strength; Rotatory strength in length form (10-40 cgs); Rotatory strength in velocity form (10-40 cgs).

Table S18. Key transitions, oscillator strengths, and rotatory strengths in the ECD spectrum of conformer **1**-8 at the CAM-B3LYP-SCRF/def2-SVP//B3LYP/6-31G(d) level of theory in MeOH with IEFPCM solvent model.

Num	transition	CI-coeff	ΔE (eV)	λ (nm)	f	Rvel	Rlen
1	49->50	0.70023	4.6487	266.71	0.7139	3.5747	8.3567
2	46->50	0.59034	5.0773	244.19	0.0105	-17.86	-18.5616
	47->50	0.27766					
3	48->50	0.63987	5.4712	226.61	0.0204	-4.8666	-4.9861
4	45->50	0.62998	6.0375	205.36	0.0181	-9.0227	-10.0007
	46->50	-0.23755					
5	46->50	-0.2413	6.3772	194.42	0.0016	1.1446	0.9682
	47->50	0.58674					
	48->50	0.26525					
6	38->50	0.26298	7.1629	173.09	0.0014	5.0017	5.5425
	41->50	0.42468					
	43->50	0.44763					
7	41->50	-0.31736	7.3411	168.89	0.0058	6.0505	6.5608
	43->50	0.39714					
	44->50	0.38581					
8	44->50	0.358	7.5897	163.36	0.1711	-12.6521	-15.2579
	49->51	0.50402					
9	41->50	-0.28003	7.6398	162.29	0.0521	12.5542	12.6598
	43->50	0.22626					
	44->50	-0.3879					
	49->51	0.42296					
10	38->50	0.41428	7.9186	156.57	0.0107	21.3603	21.7618
	39->50	0.29551					
	41->50	-0.22875					
11	40->50	0.52856	8.0086	154.81	0.1057	-44.8251	-51.4616
12	49->52	0.53696	8.0333	154.34	0.0116	16.577	17.4163
	49->53	0.32562					

13	34->50	-0.23173	8.2132	150.96	0.0022	-2.3297	-1.1625
	46->51	0.4166					
14	48->51	0.24811	8.2522	150.24	0.0528	11.053	11.8073
	48->52	-0.34339					
	49->53	0.37438					
15	40->50	-0.25894	8.3705	148.12	0.0301	13.7652	15.2361
	42->50	0.49498					
	49->54	0.30912					
16	42->50	-0.37375	8.3817	147.92	0.0759	-23.9782	-26.8527
	49->54	0.47498					
17	48->51	-0.22477	8.6677	143.04	0.0106	12.0261	11.8758
	49->54	0.24161					
	49->55	0.39521					
18	36->50	0.46785	8.6961	142.57	0.0017	0.5359	-1.3697
	37->50	0.30832					
19	39->50	0.43602	8.7642	141.47	0.0035	-2.2037	-1.2441
20	39->50	-0.29698	8.7995	140.90	0.0037	-9.4665	-6.4105
	49->55	0.37435					
	49->56	0.26995					
21	47->51	-0.27545	8.8428	140.21	0.0081	-14.1236	-6.9255
	48->51	0.30068					
22	34->50	0.43087	9.0136	137.55	0.0005	-0.985	-1.3487
	38->50	0.23929					
	39->50	-0.27304					
23	48->55	-0.26239	9.0319	137.27	0.0094	5.5403	-0.1398
	49->56	0.4199					
24	48->52	0.28109	9.1081	136.12	0.0288	-12.6197	-19.3845
25	45->51	0.24834	9.3302	132.88	0.0246	5.9239	7.6687
	48->56	-0.24546					
	49->56	0.2573					
26	45->51	0.23693	9.3455	132.67	0.0062	14.1529	10.3304
	49->57	0.53774					
27	45->51	0.40416	9.5103	130.37	0.0147	40.9045	45.4145
	48->55	-0.22834					
28	35->50	-0.31521	9.5264	130.15	0.0015	-0.6895	-0.7876
	36->50	-0.2981					
	37->50	0.38649					
29	35->50	0.31171	9.6306	128.74	0.0745	-4.507	3.2028
	48->54	0.24628					
30	35->50	0.33861	9.6824	128.05	0.0078	-25.283	-14.5274
	47->51	0.25906					

Number of the excited states; Only transitions with contribution over 10.0% were listed; Configuration-interaction coefficient; Excitation energy; Wavelength; Oscillator strength; Rotatory strength in length form (10-40 cgs); Rotatory strength in velocity form (10-40 cgs).

Table S19. Key transitions, oscillator strengths, and rotatory strengths in the ECD spectrum of conformer **1**-9 at the CAM-B3LYP-SCRF/def2-SVP//B3LYP/6-31G(d) level of theory in MeOH with IEFPCM solvent model.

Num	transition	CI-coeff	ΔE (eV)	λ (nm)	f	Rvel	Rlen
1	49->50	0.69764	4.7095	263.26	0.6824	-25.6395	-26.9513
2	46->50	0.47855	5.0460	245.71	0.0243	27.8888	28.7661
	47->50	0.30907					
	48->50	-0.35966					
3	47->50	0.45901	5.5838	222.04	0.0140	-0.9968	-1.3756
	48->50	0.49278					
4	45->50	0.365	5.8602	211.57	0.0042	5.5241	6.6053
	46->50	-0.38414					
	47->50	0.40129					
5	45->50	0.56445	6.2275	199.09	0.0178	-10.1654	-11.7174
	46->50	0.2344					
	48->50	0.28273					
6	38->50	-0.30616	7.2251	171.60	0.0014	4.3712	4.1868
	41->50	0.30178					
	42->50	0.38249					
	43->50	-0.27809					
	44->50	0.24661					
7	43->50	0.25628	7.4139	167.23	0.0112	-8.6278	-8.9813
	44->50	0.6044					
8	49->51	0.62163	7.6192	162.73	0.2389	-7.2535	-8.0426
	49->52	-0.2303					
9	42->50	0.45662	7.7036	160.94	0.0026	-0.3587	-0.3291
	43->50	0.4104					
10	38->50	0.32218	7.9695	155.57	0.0032	2.559	0.9825
	39->50	0.29777					
	41->50	0.23181					
11	40->50	-0.31324	8.0142	154.71	0.0339	57.4393	72.4563
	48->52	0.3236					
	49->52	-0.2829					
12	40->50	0.43393	8.0599	153.83	0.0919	-51.0235	-41.2956
	48->52	0.31788					
13	40->50	-0.23862	8.0903	153.25	0.0336	-5.4736	-12.1675
	49->53	0.56379					
14	34->50	0.25121	8.1857	151.46	0.0030	8.5444	2.0753
	38->50	0.36628					

	46->51	-0.3171					
15	40->50	0.25615	8.3606	148.30	0.0994	22.4716	27.3174
	49->52	-0.26323					
	49->54	0.45512					
16	37->50	0.36487	8.4610	146.54	0.0039	-3.7167	0.4181
	38->50	0.24858					
	41->50	-0.27272					
	43->50	-0.25109					
17	47->51	0.39501	8.5106	145.68	0.0184	-17.2614	-31.3338
	47->54	-0.27272					
	48->51	0.31542					
18	34->50	-0.24289	8.7630	141.49	0.0021	2.059	2.3881
	39->50	0.33322					
19	49->52	0.32437	8.8149	140.65	0.0161	5.0247	4.3508
	49->54	0.31355					
20	37->50	0.35085	8.8958	139.37	0.0092	-17.587	-20.5115
	41->50	0.29617					
	49->56	-0.2479					
21	49->56	0.52418	8.9327	138.80	0.0050	10.7566	8.6642
22	46->51	0.28419	9.0935	136.34	0.0309	-16.8783	-14.5206
	48->51	0.23619					
	49->57	0.2631					
23	47->52	0.35778	9.2302	134.32	0.0230	19.9151	20.0467
24	47->52	0.26244	9.2942	133.40	0.0397	-2.041	-1.173
	48->52	0.2623					
	49->57	0.35487					
25	48->55	-0.28966	9.3575	132.50	0.0200	5.6724	3.617
	49->55	0.4512					
	49->58	-0.23997					
26	33->50	-0.30355	9.4258	131.54	0.0047	-3.1326	-3.8603
	34->50	0.27173					
	39->50	0.35571					
27	45->51	0.35982	9.4377	131.37	0.0185	35.3278	35.4885
	48->55	0.2716					
	49->57	-0.25191					
28	47->52	0.28285	9.5637	129.64	0.0025	-8.6748	-6.9235
	47->54	-0.22989					
	47->57	-0.2705					
29	36->50	0.64482	9.6406	128.61	0.0051	-0.6934	-1.4823
30	49->58	0.2651	9.7338	127.38	0.0143	4.2357	3.3461
	49->59	0.30386					

Number of the excited states; Only transitions with contribution over 10.0% were listed; Configuration-interaction coefficient; Excitation energy; Wavelength; Oscillator strength; Rotatory strength in length form (10-40 cgs); Rotatory strength in velocity form (10-40 cgs).
